# A randomized clinical trial in preterm infants on the effects of a home-based early intervention with the 'CareToy System'

**DOI:** 10.1371/journal.pone.0173521

**Published:** 2017-03-22

**Authors:** Giuseppina Sgandurra, Jakob Lorentzen, Emanuela Inguaggiato, Laura Bartalena, Elena Beani, Francesca Cecchi, Paolo Dario, Matteo Giampietri, Gorm Greisen, Anna Herskind, Jens Bo Nielsen, Giuseppe Rossi, Giovanni Cioni, On behalf of The CareToy Consortium

**Affiliations:** 1 Department of Developmental Neuroscience, IRCCS Fondazione Stella Maris, Calambrone, Pisa, Italy; 2 Department of Clinical and Experimental Medicine, University of Pisa, Pisa, Italy; 3 Helene Elsass Center, Charlottenlund, Denmark; 4 Department of Nutrition, Exercise and Sport, University of Copenhagen, Copenhagen, Denmark; 5 Scuola Superiore Sant’Anna, Institute of Life of Sciences, Pisa, Italy; 6 Neonatal Intensive Care Unit, Pisa University Hospital Santa Chiara, Pisa, Italy; 7 The BioRobotics Institute, Polo Sant’Anna Valdera, Pontedera, Pisa, Italy; 8 Department of Neonatology, Copenhagen University Hospital (Rigshospitalet), Copenhagen, Denmark; 9 Department of Neuroscience and Pharmacology, University of Copenhagen, Copenhagen, Denmark; 10 Unit of Epidemiology and Biostatistics, Institute of Clinical Physiology, CNR, Pisa, Italy; TNO, NETHERLANDS

## Abstract

CareToy system is an innovative tele-rehabilitative tool, useful in providing intensive, individualized, home-based, family-centred Early Intervention (EI) in infants. Our aim was to evaluate, through a Randomized Clinical Trial (RCT) study, the effects of CareToy intervention on early motor and visual development in preterm infants. 41 preterm infants (range age: 3.0–5.9 months of corrected age) were enrolled and randomized into two groups, CareToy and Standard Care. 19 infants randomized in CareToy group performed a 4-week CareToy program, while 22 allocated to control group completed 4 weeks of Standard Care. Infant Motor Profile (IMP) was primary outcome measure, Alberta Infant Motor Scale (AIMS) and Teller Acuity Cards were secondary ones. Assessments were carried out at baseline (T0) and at the end of CareToy training or Standard Care period (T1). T1 was the primary endpoint. After RCT phase, 17 infants from control group carried out a 4-week CareToy program, while 18 infants from the CareToy group continued with Standard Care. At the end of this phase, infants were re-assessed at T2. In RCT phase, delta IMP total score and variation and performance sub-domains were significantly higher (P<0.050) in CareToy group if compared to Standard Care group. Similar results were found for Teller Acuity Cards, while no differences between groups were found for AIMS. No differences were found in any outcome measure results (T2-T0), between infants who started CareToy training before or after one month of standard care. This RCT study confirms the results of a previous pilot study, indicating that CareToy system can provide effective home-based EI.

**Trial Registration:** This trial has been registered at www.clinicaltrials.gov (Identifier NCT01990183).

## Introduction

Prematurity is a risk factor for long-term visual, cognitive and psychosocial impairments [1]. Evidence suggests a direct causative link of poor visual function, motor delay, neurodevelopmental disorders (NDDs) with prematurity and prolonged recovery in Neonatal Intensive Care Unit (NICU) [2,3].

In recent years, interest in early intervention (EI) has increased considerably with the awareness that it may be pivotal in improving neurodevelopmental outcome for infants at risk for NDDs [4].

EI implies that intervention should start as soon as possible in order to prevent emergence of disorders and promote better functional outcomes [5–7]. EI focuses on promotion of milestone attainment across all developmental domains, to support harmonic development. A number of systematic and Cochrane Reviews [8–11] have reported that EI has positive effects on development of cognitive and motor functions, especially if EI is based on key factors such as child-initiated movement, task specificity, parent-engagement and environmental modification. However, these studies have also pointed out that effectiveness of EI programs has not yet been fully proven, due to extreme heterogeneity among programs with respect to intensity, focus, setting, and participants.

One solution may be to create a common family-centered, home-based setting, suitable in obtaining quantitative measures of infant activity and progress through remote monitoring and management by rehabilitation staff. In this context, the CareToy System (www.caretoy.eu), which has been previously described [12–14] may satisfy these requirements. The CareToy system is an innovative tele-rehabilitative tool, useful in providing intensive, individualized, home-based and family-centred EI in infants. It is a smart system based on a common-component baby gym, composed of three modules: 1) an instrumented baby gym with mechatronic hanging toys, which measure and stimulate movements, 2) a vision module which measures and promotes attention and gaze movements, 3) a sensorized mat which measures and promotes postural control. Each module also incorporates a built-in signal processor, memory and wireless communication which connects the system to rehabilitation staff.

CareToy training is inspired by the enriched environment concept [15] and based on goal-directed activities aimed at encouraging infants to perform specific tasks. Training may be remotely monitored and tailored by rehabilitation staff according to specific developmental needs of each infant, while parents actively play with their child.

A previous pilot study confirmed both feasibility of the CareToy System as a tool in promoting EI in infants and validity of study design [16–17]. This Randomized Clinical Trial (RCT) study was aimed at testing short-term effects of CareToy training on motor and visual development in 3–9 month old preterm infants.

## Patients and methods

### Study design

This was a randomized, multicenter, evaluator-blinded, RCT, designed according to CONSORT Statement [18,19] (S1 Table). At first, it was orginal designed as cross-over trial but after chosing T1 as the primary endpoint (see later) the trial was modified to a parallel RCT followed by an open phase. Eligible infants were identified by the two clinical centres involved in the study: IRCCS Fondazione Stella Maris with NICU of Pisa University Hospital in Italy and Helene Elsass Center with the University of Copenhagen in Denmark. Detailed information about the CareToy Project were provided to attending families. If parental written consent was obtained, infants were enrolled. Perinatal and clinical data were collected upon enrolment, and infants were evaluated during their first months through standard neurological examinations. At 3 months of corrected age, infant gross-motor abilities were quantified by the Ages & Stage Questionnaire Third Edition (ASQ-3) [20] gross motor area, filled out by parents, in order to define the most appropriate starting point for each infant.

After baseline evaluation (T0), infants were randomly assigned to either CareToy group or Standard Care group. Twins were allocated to the same group. All infants were re-assessed at T1 (4 weeks post T0), on primary and secondary outcomes. After this RCT phase, according to study design, infants who started out with Standard Care at T1 were given the opportunity to carry out a 4-week CareToy program, while infants that had already performed CareToy training switched over to Standard Care. 4 weeks after this phase all infants were re-assessed (T2). A further assessment is planned at 18 months of corrected age (T3), but the results of this timepoint are not included in this paper. All assessments were recorded on video and scored off-line by expert therapists, blinded to group allocation.

The study was conducted according to Good Clinical Practice and Declaration of Helsinki principles and supervised by an Ethics Advisory Board. Approval was obtained by the Ethics Committee of the Hovedstaden Region (Denmark) in June 2012, Ethics Committee of Pisa University Hospital (Italy) in June 2013, Tuscan Region Pediatric Ethics Committee (Italy) in February 2014 and IRCCS Fondazione Stella Maris Review Board in May 2014. The first infant was enrolled in the pilot study in July 2013, but stopped afterwards due to CareToy technical adjustments and restarted in November 2013 when the protocol was registered. The authors confirm that the trial has been registered at ClinicalTrials.gov (NCT01990183, https://clinicaltrials.gov/ct2/show/NCT01990183).

### Study populations

As previously described [12], eligible infants were preterm infants with the following characteristics:

- born between 28 + 0 and 32 + 6 (weeks + days) of gestational age
- aged 3–9 months of corrected age who had achieved a predefined cut-off score in gross motor ability derived from ASQ-3 [20].

Exclusion Criteria were:

- birth weight below the 10th percentile;
- brain damage i.e. intra-ventricular haemorrhage < grade 1, any degree of periventricular leukomalacia, or brain malformation;
- any form of seizure;
- severe sensory deficits (blindness, deafness);
- other severe non-neurological malformations;
- participation in other experimental rehabilitation studies.

### Intervention conditions

#### CareToy intervention

CareToy intervention (detailed in Sgandurra et al [12,16]) is an intensive, highly customized, home-based, family-centred training program, provided through remote management of a CareToy system delivered at home. It consists of specific goal-directed activities, called scenarios, remotely planned by the clinical/rehabilitative staff according to specific infant needs and capabilities. Training is multiaxial with a high degree of variability and complexity. Each scenario lasts from 2 to 10 minutes and promotes different aspects of motor and visual development, such as head rotation and gaze movement, grasping and eye-hand coordination. Based on postural control and rehabilitative needs, activities integrated in CareToy system can be variably planned in supine, prone or sitting position. Training is programmed daily for 30–45 minutes for 4 weeks including weekends (a total of 28 days). According to general guidelines previously developed during pilot study [16], CareToy training at home is organized into two phases. The first one, lasting a week, is devoted to infant and parent habituation to system and identification of main rehabilitation goals. The second phase, lasting three weeks, consists of continuous planning and customization of training in relation to daily activities and progress. At the end of each day, the CareToy System automatically sends a report of training to rehabilitative staff, so that it can be monitored and directed to promote progressively more complex abilities when the previous ones have been achieved.

#### Standard care

As previously described [12,16], Standard Care consists of a bimonthly follow-up check, during which current care advice on the early management of preterm infants and booklets dedicated to home-care of preterm infants are distributed, according to the standard recommandations of the two involved countries (Italy and Denmark). In rare cases, sporadic sessions with a physical therapist for special assistance were arranged. In such cases, the number and type of performed activities were recorded.

### Measures

Due to the characteristics of CareToy intervention which mainly address goal-directed motor activities, a motor scale as primary outcome measure was chosen. In particular, the Infant Motor Profile (IMP), previously indicated as secondary outcome, was shifted to the primary one. The study protocol, as registered at Clinicaltrials.gov, was modified accordingly and published in the description of study protocol [12] and in the results of pilot study [16] (S1 Text and S2 Text). IMP is a recent test aimed at assessing motor behaviour of preterm and term infants aged 3–18 months [21–23]. It consists of 80 items scored off-line on the basis of standardized video recordings. Total IMP score constitutes the mean of five subdomains (variation, adaptability, symmetry, fluency and performance). Adaptability is only scored for infants older than 6 months, so it was not included in this study. This evaluation was carried out at T0, T1 and T2.

Secondary outcome measures consisted of one for motor and one for visual assessments. The first one was the Alberta Infant Motor Scale (AIMS). It is a standardized scale, used in infants from term until 18 months of age [24]. It assesses infant motor abilities and quality of posture and movement in four positions: prone, supine, sitting and standing. It is possible to determine a total overall score and subscores for each assessed position. AIMS evaluation was performed at T0, T1 and T2 [25]. At the same time points, Teller Acuity Cards were used to evaluate visual acuity. It is based on assessment of infant attention to a series of cards showing stripes of different widths. This tool allows for a rapid assessment of visual acuity (grating) in infants and young children and other populations where verbal response to recognition of visual acuity charts (letters) is difficult or impossible [26]. It evaluates development of visual acuity and has been used in several studies for diagnostic purposes and to assess results of early intervention [27, 28].

### Statistical analyses

Sample size was calculated on the basis of previous results on IMP [16] and on design of a parallel RCT study. 38 infants (42 in total, including drop-outs) were needed to detect a clinically relevant change of 2.4 points with a power of 80% at a significance level of 0.05.

Clinical data were analyzed by means of Statistical Package for Social Sciences (SPSS, version 20.0). Normality of distribution was verified by Shapiro-Wilk’s test.

To test a-priori baseline differences between the two groups for characteristics (Gestational age, Birth weight, Gender, Corrected Age) and baseline measures (IMP total and subdomains, AIMS total and subdomains and Teller) the t-test for unrelated samples and non-parametric Mann-Whithney test were used for normal and non-normal distributed data, respectively.

Programmed and executed days and hours of CareToy training and their rate were calculated in order to assess compliance with CareToy intervention. In particular, rates were calculated by computing percentages of ratio between executed and programmed days and hours, respectively.

Changes in primary and secondary outcome measures were calculated between baseline (T0) and post-intervention period (T1). In order to verify effect of CareToy training versus Standard Care on primary endpoint (T1-T0), the t-test for unrelated samples and the non-parametric Mann-Whithney test were used for normal and non-normal distributed scores, respectively. Effect size was computed using Cohen’s d for IMP total, AIMS total and Teller. Commonly used criteria specify that a value below 0.2 is regarded as no effect, a value of 0.2–0.5 as a small effect, a value of 0.5–0.8 as a medium-sized effect and a value above 0.8 as a large effect [29].

Moreover, a Pearson correlation analysis was carried out between AIMS total delta scores (T1-T0) and IMP total and IMP performance delta scores (T1-T0), respectively, in order to detect if there were any correlations between the two motor outcome measures, as detected in the validation studies of IMP [21–23].

A further analysis was planned to evaluate whether CareToy training carried out one month after of standard care (T1) may have different effects than CareToy performed at the beginning (T0). In particular, we compared the delta of T2-T0 between the two groups.

Finally, correlations between delta changes after CareToy training in primary and secondary outcome measures that showed statistical significant changes and hours of CareToy training performed were determined by linear regression analysis (Pearson correlation coefficient). Non-adjusted significance level for all analyses was set at p<0.05.

## Results

### Participants

**Fig 1:**
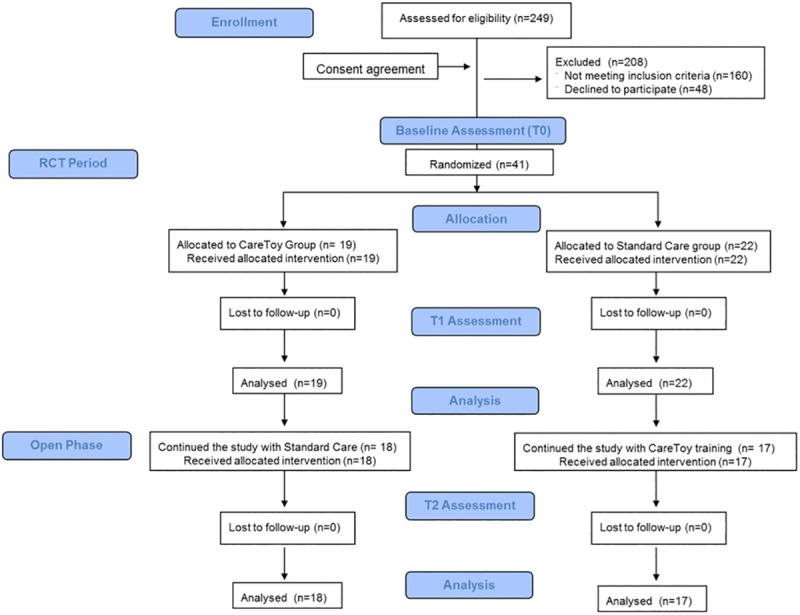
Flow-chart of study according to CONSORT diagram.

249 infants were assessed for eligibility. 208 were excluded from study because they did not meet inclusion criteria (160) or families declined to participate (48) (Fig 1).

41 infants (mean age 3.9 ± 0.8 months, range: 3.0–5.9) were recruited between May 2014 and April 2015 and 19 infants (mean age 3.6 ± 0.4 months, range: 3.1–4.9) were randomly allocated to CareToy and 22 (mean age 4.1 ± 1.0 months, range: 3.0–5.9) to Standard Care. Baseline characteristics of participants are reported in Table 1. No statistically significant differences were found between the two groups at baseline.

**Table 1:** 10 Main characteristics of the CareToy and Standard Care groups at baseline (T0).

Characteristic		CareToy group (n = 19)	Standard Care group (n = 22)	*p value*^*^*^
Gestational age (weeks), mean (SD)		30.7 (1.4)	30.82(1.1)	0.772 ^*^
Birth weight (grams), mean (SD)		1368.3 (330.3)	1459.5 (275.6)	0.308^§^
Gender M/F		8/11	11/11	
Corrected Age (months), mean (SD)		3.6 (0.4)	4.1 (1.0)	0.704^§^
ASQ-3 Gross Motor Area				
	Form 2 (median)	n = 3/19 (40)	n = 2/22 (52.5)	*-*
	Form 4 (median)	n = 16/19 (50)	n = 16/22 (50)	*-*
	Form 6 (median)	-	n = 4/22 (42.5)	*-*
Twins		8/19	10/22	
Infant Motor Profile, mean (SD)				
	Total	69.9 (3.5)	69.8 (3.4)	0.924^*^
	Performance	45.9 (6.6)	48.1 (7.4)	0.239^§^
	Variation	64.9 (5.2)	63.6 (3.0)	0.820^§^
	Fluency	75.0 (0.0)	75.0 (0.0)	1.000^§^
	Symmetry	93.8 (8.1)	92.5 (7.8)	0.387^§^
AIMS, mean (SD)				
	Total	10.8 (3.6)	11.7 (3.3)	0.316^§^
	Prone	3.5 (1.4)	3.9 (1.5)	0.583^§^
	Supine	4.4 (1.0)	5.0 (1.3)	0.121^§^
	Sitting	1.7 (1.6)	1.5 (1.0)	0.731^§^
	Standing	1.2 (0.5)	1.3 (0.4)	0.445^§^
Teller Acuity Card (cy/degree), mean (SD)		2.6 (1.0)	3.2 (1.2)	0.158^§^

Abbreviations: n: number; SD: Standard Deviation

^***^***^ Significant non-adjusted level <0.050

^*^Independent t test

^§^ Mann-Whitney

All infants underwent 4 weeks of intervention according to their allocation. Infants allocated to the CareToy group received a mean of 25.6 ± 3.1 days and 10.7 ± 2.1 hours of training with a ratio to programmed days and hours of 95.1% and 72.1%, respectively. None of the infants of CareToy or Standard Care group received any special sessions with a physical therapist. All infants were reassessed the week after the end of intervention period (T1).

After this RCT period, 17/22 infants previously allocated to the Standard Care group carried out CareToy training for 4 weeks (mean of 21.9 ± 4.2 days and 9.9 ± 3.6 hours with a ratio to the programmed days and hours of 95.6% and 79.6%, respectively) and were reassessed at T2. At the same time point (T2), 18/19 infants previously allocated to the CareToy group were re-evaluated after the Standard Care period.

Considering the whole group of 26 infants who carried out the CareToy intervention in the RCT period (19) and in the open phase (17) the infants received a mean of 25.1 ± 4.0 days and 13.7 ± 3.8 hours of training with a ratio to programmed days and hours of 95.3% and 75.3%, respectively.

### Primary outcome measure (IMP)

After 4-week intervention period, delta IMP total score (change from T0 to T1) was significantly higher in CareToy group compared to Standard Care group (Table 2). Moreover, there were also significant changes for Performance and Variation subdomains. The Cohen’s d value was 0.69.

**Table 2:** Mean score increase from before 4-week intervention to after (T1-T0).

		CareToy group (n = 19)	Standard Care group (n = 22)	*p value*^*^*^
		Delta mean (SD)	Delta mean (SD)	
**Primary outcome measure**				
IMP				
	Total	6.0 (2.2)	4.3 (2.7)	0.039^*^
	Performance	10.6 (4.3)	7.3 (5.0)	0.028^*^
	Variation	8.3 (6.6)	6.9 (5.2)	0.044^*^
	Fluency	1.1 (3.4)	0.00 (0.00)	0.123^§^
	Symmetry	3.7 (6.6)	4.4 (6.0)	0.440^§^
**Secondary outcome measures**				
AIMS				
	Total	4.8 (3.1)	3.9 (2.7)	0.308^*^
	Prone	2.2 (2.1)	1.8 (1.5)	0.749^§^
	Supine	1.7 (0.9)	1.0 (1.0)	0.077^*^
	Sitting	0.5 (0.8)	0.6 (1.4)	0.669^§^
	Standing	0.4 (0.5)	0.4 (0.5)	0.939^§^
Teller Acuity Card (cy/degree)		2.0 (1.2)	1.2 (1.4)	0.035^*^

Abbreviations: n: number; SD: Standard Deviation

^***^***^ Significant level <0.050

^*^Independent t test

^§^ Mann-Whitney test

There were no differences in changes between the two groups from T2 to T0 (Table 3).

**Table 3:** Mean increase of T2-T0 in the two groups.

		CareToy + plus SC (n = 18)	Standard Care + CareToy (n = 17)	*p value*^*^*^
		Delta mean (SD)	Delta mean (SD)	
**Primary outcome measure**				
Infant Motor Profile				^*^
	Total	9.5 (3.1)	9.2 (3.6)	0.091^*^
	Performance	15.9 (5.0)	15.4 (4.9)	0.077^*^
	Variation	14.1 (6.6)	14.3 (5.5)	0.092^*^
	Fluency	1.1 (3.4)	0.5 (2.0)	0.563^§^
	Symmetry	3.9 (7.6)	7.1 (9.5)	0.115^§^
**Secondary outcome measures**				
AIMS				
	Total	9.4 (4.0)	9.6 (3.2)	0.870^*^
	Prone	4.1 (2.7)	4.5 (2.0)	0.615^*^
	Supine	2.3 (1.1)	2.6 (0.9)	0.380^*^
	Sitting	2.2 (1.8)	1.8 (1.6)	0.586^§^
	Standing	0.8 (1.0)	0.5 (0.5)	0.273^§^
Teller Acuity Card (cy/degree)		3.2 (1.3)	3.5 (1.4)	0.361^*^

Abbreviations: n: number; SD: Standard Deviation

^***^***^ Significant level <0.050

^*^Independent t test

^§^ Mann-Whitney test

### Secondary outcome measures

#### AIMS

After 4-week intervention period, change was higher (but not statistically significant) in CareToy group compared to Standard Care for AIMS total score and also for supine and prone subdomains (Table 2). Cohen’s d value of AIMS total was 0.33.

Pearson correlation analysis showed a significant correlation between changes in AIMS total delta scores and IMP total (0.572, p<0.0001) and IMP performance (0.597, p<0.0001) delta scores of T1-T0.

There were no differences in changes between the two groups from T2 to T0 (Table 3).

#### Teller acuity cards

After 4-week intervention period, there was a significantly greater improvement in visual acuity in CareToy group compared to Standard Care group (Table 2) with a Cohen’s d value of 0.68.

There were no differences in changes between the two groups from T2 to T0 (Table 3).

### Regression analysis

**Fig 2:**
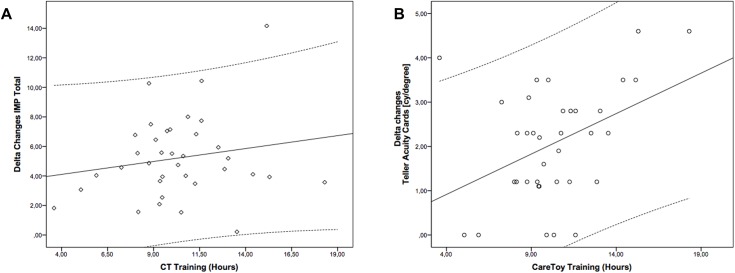
Regression analysis. Legend. Scatter plot of delta changes after CareToy training in Infant Motor Profile (A) and in Teller Acuity Cards versus number of hours of performed CareToy training. Solide lines represent the linear fit of data. Dotted lines represent Confidence Interval at 95%.

Regression analysis carried out immediately after training showed a significant relationship between delta changes in Teller scores and amount of performed CareToy training (r = 0.411, p = 0.013) while it was not significant for delta changes in IMP scores (r = 0.183, p = 0.285) (Fig 2).

## Discussion

Motor and visual development are frequently delayed in first months of life in preterm infants. EI programs are aimed at minimizing these developmental delays. In this framework, the main goals of CareToy training are to promote postural control, reaching, grasping, visual attention and orientation in highly variable and tailored activities set at various levels of complexity.

Results of RCT period (T1 versus T0) indicate that the 4-week EI program provided through CareToy system can significantly improve motor and visual development in preterm infants. In the motor domain of the primary outcome measure (IMP), we found medium-sized, statistically significant effects on IMP total scores compared to Standard Care. The effect appeared to be driven by improvements in variation and performance subdomains. Variation is brought about by the explorative activity of nervous system and is used for shaping it, reflecting the interaction between individual motor repertoire and environment. A high variation indicates an abundance in cerebral connectivity in typical development, while a more limited variation can be accounted for by structural anomalies in which disturbances of cortical connectivity may play a prominent role [29]. Enlargement of limited movement repertoire is an aim of EI, even if animal data indicate that this is difficult to achieve [30]. To our knowledge, EI through CareToy is the first program that has demonstrated significant effects, when formally compared to Standard Care, in enhancing variability of motor behavior. In addition, CareToy EI is able to promote an earlier achievement of developmental motor milestones evaluated by the IMP performance subdomain. Fluency (i.e. ability to fine-tune motor output) and symmetry (i.e. presence or absence of stereotyped asymmetries) subdomains were not significantly different in CareToy group if compared to Standard Care one. However, the CareToy seems to be able to enhance fluency, whereas no changes were detected in Standard Care group. Although CareToy system has the ability to stimulate goal-directed activities for asymmetric conditions, the lack of effect on symmetry scores is likely explained by high symmetry scores at baseline, indicating that the sample of infants of this study did not need this kind of intervention.

Regarding the secondary motor outcome measure (AIMS), we found only a slight effect in the total score, with higher changes in supine and prone scores in the CareToy group compared to the Standard Care one. These two positions, supine and prone, were the most trained ones in relation to the age window and developmental needs of enrolled infants. Lack of significant effects of Care Toy training on AIMS measures, unlike IMP, could be related to the structure of AIMS items and relative scoring. Each item within the “motor window” of AIMS is scored as “observed” or “not observed”, so minor changes cannot be specified. In addition, AIMS only assesses gross motor development while IMP evaluates both gross and fine motor development. Another aspect could be related to the different psychometric properties of the two scales and limitations for detecting short term effects by AIMS, as already pointed out in literature [31]. In any case, a significant correlation was found between changes in AIMS total scores and IMP total and performance scores.

A further interesting result is that EI provided through the CareToy system is able to significantly promote visual acuity as assessed by Teller Acuity cards, similar to other EI programs in preterm infants, such as infant massage therapy [27]. This finding is probably related to the high sensitivity of visual development in the first months, characterized by rapid maturation of visual cortex and visual acuity. Promotion of visual development is particularly crucial in preterm infants because a significant number show delayed maturation of visual acuity, compared to their term-born peers, with further deficits in domains of visual perception and visual-motor integration [32]. Thus, early promotion of visual acuity can have a positive effect also on these more complex visual abilities. Moreover, considering the whole group of infants that performed CareToy training, we found a positive correlation between visual acuity changes and number of hours of training, while no correlation was found for IMP changes. This finding could be related to differences in dosage response between visual and motor system, as well documented in literature, where a relationship between dosage and effects has been found for the treatment of amblyopia [33] and no relationship between dosage and effects for motor treatment. [34,35]. Moreover, our finding could be related to the type of measurement and type of training. The goal-directed motor activities promoted in CareToy training are largely dependent on the character and preferences of each infant (e.g. from early reaching on midline to grasping while rolling) and IMP scale is able to capture several aspects of motor development. This variability can justify why we found improvement only in the whole group for IMP scores. On the contrary, Teller Acuity cards evaluate a specific aspect of visual development, i.e. visual acuity, that was specifically trained for in all infants using visual stimuli (e.g. videos on screen, lights on arch, on toys and on feedback walls).

Moreover, no differences were found for any of the outcome measures, when comparing the results of CareToy training carried out before or after one month of standard care. These results justify the ethical decision of offering CareToy training to those infants who were randomly allocated in the Standard Sare group. All these data confirm the hypothesis that CareToy training when performed for 4 weeks in the very first months is able to have at least a short term impact on motor and visual development. The choice to have an open phase after the RCT period, creditable from an ethical point of view, hampers the evaluation and extrapolation of longer term effects of CareToy training.

A strong relation has been reported between visual and motor functions in the first months of life and infant development in the other domains [36] and long-term neurodevelopmental outcomes of newborn infants at risk [37]. Moreover, results of RCTs and systematic reviews [8–11], indicate that EI has positive effects on cognitive and motor development, and hopefully could reduce prevalence of neurodevelopmental disabilities and improve quality of life.

## Conclusions

This RCT study has strengthened evidence that the CareToy system can provide effective home-based, individualised EI, at least in preterm infants without severe medical complications. CareToy intervention is a strong candidate for testing on a larger scale with the aim of achieving long-term benefits to children with increased risk of developing neurological impairments. Another important aspect of our approach, in relation to its transferability to clinical practice, deals with cost of this technology, when ready for industrialization. Recently, an early-stage economic model [38] for the evaluation of these aspects has been developed.

## Supporting information

S1 Table CONSORT checklist. (DOC)

S1 Text Italian clinical protocol _Italian version. (DOC)

S2 Text Italian clinical protocol _English version. (DOC)
