# Supplementary material for: A randomized clinical trial in preterm infants on the effects of a home-based early intervention with the 'CareToy System'
Source: PLoS One. 2017 Mar 22;12(3):e0173521. doi: 10.1371/journal.pone.0173521 (PMC5362053; doi:10.1371/journal.pone.0173521)
Supplement: S1 Text — (DOC) [file pone.0173521.s002.doc]

**SCHEDA RIASSUNTIVA DI PROTOCOLLO DI STUDIO CLINICO**

| Elementi identificativi | | | |
| --- | --- | --- | --- |
| Titolo dello studio | “CareToy: un nuovo sistema modulare intelligente basato su giochi biomeccatronici per la riabilitazione a domicilio dei bambini” | | |
| Versione e data del protocollo | Versione del Codice della Ricerca: CareToy ICT-STREP proposal number 287932 | | |
| Documenti  (versione e  data) | Lettera di presentazione (versione 1.0 del 20 giugno 2013)  Allegato A: Case Report Form (IT)  Allegato A1: Prospetto protocollo di valutazione (IT)  Allegato B: Prospetto informativo per il paziente (IT)  Allegato C: Modulo di consenso per il paziente (IT)  Allegato D: Lettera informativa per il medico (IT)  Allegato E1: Sinossi sulle Specifiche tecniche del CareToy H (IT)  Allegato E2: Deliverable MS-2 (Descrizione tecnica delle componenti de3l CareToy) (EN)  Allegato E3: Prove per dispositivo (preventivo Elettra) (IT)  Allegato F1: Nota Informativa Assicurazione  Allegato F2: Polizza Assicurativa  Allegato F3: Quotazione Assicurazione  Allegato G: CareToy Consensus Agreement (EN)  Allegato H: Ethical issues (Deliverable 7.3) (EN)  Allegato I: Protocollo di studio Maggio 2013 (Deliverable 7.1) (EN)  Allegato L: Progetto di ricerca completo approvato dall'Unione Europea (EN)  Allegato M: Curriculum sperimentatore (IT) | | |
| Codici | *OSC EUDRACT:*  NA | *Promotore della sperimentazione:* | *FSM:*  ………./2013 |

| Informazioni amministrative | | | |
| --- | --- | --- | --- |
| Sperimentatore principale, denominazione e sede del centro coordinatore | | | Sperimentatore principale in Italia:  Prof. Giovanni Cioni  IRCCS Fondazione Stella Maris  Viale del Tirreno 341/ ABC  56128 Calambrone (Pisa) – Italia  Altro centro sperimentatore europeo (Danimarca)  Helene Elsass Center Holmegårdsvej 28 2920 Charlottenlund  Copenhagen – Denmark  Centro coordinatore:  The BioRobotics Institute - Scuola Superiore Sant'Anna Polo Sant'Anna Valdera  Viale Rinaldo Piaggio 34 56025 Pontedera (Pisa) – Italia |
| Denominazione del promotore della sperimentazione | | | Prof. Giovanni Cioni  IRCCS Fondazione Stella Maris  Viale del Tirreno 341/ ABC  56128 Calambrone (Pisa) – Italia |
| Lettera di intenti dello sperimentatore | | | Vedi lettera |
| Elenco dei centri coinvolti | | | - BioRobotics Institute – Scuola Superiore Sant’Anna, Italia - IRCCS Fondazione Stella Maris, Italia - STMicroelectronics SRL, Italia - Univerza V Ljubljani, Slovenia - Universitaet Hamburg, Germany - Fonden for Helen Elsass Center, Denmark - Marketing Research & Development SPA, Italia |
| Date inizio-fine studio | | | Inizio-Fine Studio Clinico: Novembre 2013 – Aprile 2015 |
| Assicurazione | | | L’IRCCS Fondazione Stella Maris ha prima provveduto a contattare il broker ottenendo la quotazione specifica (vedi Allegati F1, F2 ed F3) per il progetto CareToy da sottoscrivere appena i prototipi finali del CareToy fossero stati prodotti e iniziato il reclutamento.  L’IRCCS Fondazione Stella Maris ha provveduto a stipulare la polizza assicurativa con decorrenza dall’1/11/2013 al 31/10/2014 (allegato 4) |
| Convenzione economica | | | Il progetto CareToy è finanziato dalla Comunità Europea. |
| Proprietà dei dati | | | Come verrà specificato in seguito (vedi Reclutamento), l’IRCCS Fondanzione Stella Maris a tutela della privacy e dell'anonimato assegnerà a ciascun bambino al momento del reclutamento un codice numerico che verrà conservato in forma separata in modo che il database non conterrà nessun dato demografico e clinico. L'accesso a tali dati sarà limitato al solo personale dell’Istituto direttamente coinvolto nello studio mentre tutti i dati verranno trattati in forma anonima.  I dati del progetto trattati in forma generale saranno proprietà di tutti i membri del Consorzio CareToy come da Consensus Agreement sottoscritto da tutte le parti (vedi Allegato G). |
| Fornitura farmaci | | | Nel disegno dello studio non è previsto l'impiego di farmaci |
| Dati scientifici dello studio | | | |
| Fasi e disegno | | Lo studio sperimentale verrà effettuato in due fasi. Inizialmente verrà condotta una fase pilota che prevede l’applicazione, su un piccolo gruppo di bambini (max 10), del protocollo di studio finalizzato all’ottimizzazione del funzionamento del sistema CareToy H dal punto di vista tecnico (nel software dei giochi e dei protocolli di trasmissione e di analisi dei dati). La fase pilota prevede prima la sperimentazione del Caretoy (training sperimentale) per 4 settimane e poi il periodo di controllo di uguale durata.  In seguito verrà avviato un trial clinico sperimentale randomizzato (RCT) di tipo multicentrico e con disegno cross-over [figura 1] nel quale verranno comparati gli effetti del programma di intervento con il sistema CareToy Home (H) (training sperimentale) con la standard care.  L’arruolamento e la sperimentazione clinica per entrambe le parti (fase pilota ed RCT) avverrà parallelamente in due centri clinici, in Italia presso la Fondazione Stella Maris e in Danimarca presso l’ Helen Elsass Center.  Lo studio RCT è stato disegnato con cross-over per dare l'opportunità, a tutti i bambini arruolati di effettuare un training con il sistema CareToy H.  La durata complessiva del progetto è di 3 anni, di cui un anno sarà dedicato alla sperimentazione clinica.    Training sperimentale: Il training con il CareToy prevede l'uso quotidiano (30-40 minuti al giorno) per 5 giorni a settimana per 4 settimane consecutive del Sistema CareToy H, un box bio-meccatronico e multisensoriale, ideato come sistema modulare "intelligente" che impiega innovative strategie tecnologiche che consentono di rilevare, registrare e quantificare le attività svolte dal bambino all'interno del box fornendo dati sulle competenze visuo-attentive, posturo-motorie e sulla funzione manuale; (per dettagli vedi dispositivo medico).All'interno del CareToy H vengono proposte al bambino attività psico-motorie personalizzate ed individualizzate finalizzate alla promozione dello sviluppo psicomotorio.Le attività di "gioco" quotidiane svolte all’interno del sistema CareToy H sono suddivise in base anche alla disponibilità del bambino e dei suoi genitori in 3 sessioni della durata di circa 10 minuti ciascuna; tali sessioni potranno essere ulteriormente frazionate nel corso della giornata. Il sistema, infatti, sfruttando l'impiego della Tele-riabilitazione, consente di effettuare il training presso il domicilio, ma sotto monitoraggio da parte dello staff riabilitativo (neuropsichiatri infantili e terapisti della neuropsicomotricità dell’età evolutiva); Il bambino utilizzerà quindi il CareToy H in casa propria ma i dati, acquisiti durante la seduta di training, verranno automaticamente caricati su di un server collegato al Centro Clinico Riabilitativo di riferimento e così lo staff riabilitativo potrà monitorare "da remoto" le attività programmate, i progressi e di conseguenza riprogrammare le nuove attività da proporre in base alle esigenze di sviluppo del bambino. La famiglia sarà istruita all'uso del CareToy H nei giorni precedenti alla consegna del sistema attraverso un training parentale e usufruirà di una assistenza clinica e tecnica domiciliare nella prima settimana di training e, se necessario, nelle settimane successive. Prima e dopo il periodo di training sperimentale/controllo i bambini verranno valutati secondo tempi stabiliti (vedi Timeline) con scale cliniche e con un sottosistema ingegnerizzato del CareToy chiamato CareToy Clinical (C) messo a punto per la valutazione quantitativa del grasping e dell’attenzione visiva (vedi misure di outcome e descrizione del dispositivo).  **TIMELINE:**  Il campione, durante il periodo di studio dell’RCT, sarà valutato in 4 tempi differenti: T0, T1, T2, T3 (vedi tabella 1). Le valutazioni in Italia si svolgeranno presso l'IRCCS Stella Maris, Calambrone ed impegneranno i bambini ed i genitori per 2 giorni.   - T0: nella settimana precedente all'avvio del training - T1: nella settimana successiva al primo periodo di training sperimentale/standard - T2: nella settimana successiva al secondo periodo di training standard/sperimentale (cross-over) - T3: 18 mesi dopo la fine dei training.   Nella tabella 1 sono riportati i tempi di utilizzo delle scale di valutazione e dei questionari.  La fase clinica dello studio può essere schematicamente suddivisa in: A) FASE 1 (pre-T0):Identificazione dei casi reclutabili L'eleggibilità del campione, in accordo con i criteri di inclusione/esclusione, sarà valutata dal team del Dipartimento di Neonatologia dell'Azienda Ospedaliera Pisana-Ospedale Santa Chiara.  Ai genitori dei bambini eleggibili al momento della dimissione sarà esposto il progetto in generale e se esprimeranno il loro interesse verranno contattati dall’equipe dell’IRCCS Fondazione Stella Maris che esporrà dettagliatamente il progetto di ricerca e, se la famiglia sarà interessata a partecipare allo studio, si provvederà alla compilazione dei moduli di consenso informato. Reclutamento Solo dopo aver dopo aver ottenuto il consenso scritto alla partecipazione allo studio da parte dei genitori, il bambino sarà reclutato.  Durante il reclutamento, tramite colloquio con i genitori e la presa visione dei documenti clinici, verrà compilato il CRF (vedi allegato) e saranno acquisiti e registrati, in un apposito database i dati clinici (sesso, età gestazionale), i dati relativi alla storia prenatale (madre primipara/pluripara, gravidanza a rischio es. per età materna avanzata, diabete gestazionale, ipertensione...), perinatale (tipologia di parto, peso alla nascita, APGAR score, dettagli della storia clinica perinatale) e i risultati degli esami di screening e/o di eventuali ulteriori esami strumentali (ad es. ecografia cerebrale). Inoltre verrà effettuato un esame clinico generale e neurologico del bambino e se al momento del reclutamento il bambino avrà un’ età corretta inferiore alle 20 settimane, sarà effettuata anche la valutazione della motricità spontanea con video registrazione secondo il metodo **Prechtl's (Qualitative Assessment of General Movements).** A tutela della privacy e dell'anonimato a ciascun bambino sarà assegnato inizialmente un codice numerico che verrà conservato in forma separata in modo che il database non conterrà nessun dato demografico e clinico. L'accesso a tali dati sarà limitato al solo personale locale direttamente coinvolto nello studio mentre tutti i dati verranno trattati in forma anonima.  Dopo il reclutamento ai genitori sarà chiesto di compilare un questionario (*Ages & Stages Questionnaire)* sullo sviluppo del bambino con cadenza mensile allo scopo di valutare, sulla base delle abilità grosso-motorie acquisite nel corso dei primi mesi di vita, il raggiungimento di uno score minimo (vedi criteri di inclusione e misure di outcome) ed individuare per ciascun bambino, il momento più opportuno per l'entrata nella fase sperimentale.  **- FASE 2 (T0): Valutazione al baseline e randomizzazione**  La valutazione al baseline sarà effettuata quando il bambino avrà una età corretta compresa tra 2 e 9 mesi ed avrà acquisito le competenze grosso-motorie in accordo con i criteri di inclusione (punteggio a *Ages & Stages Questionnaire, vedi criteri di inclusione*).  La valutazione di baseline prevede l'impiego di specifiche scale di sviluppo e la compilazione di questionari da parte dei genitori (*per dettagli vedi "misure di outcome").*  Dopo la valutazione il campione arruolato per l’RCT sarà allocato, in modo *random* da un computer generatore di sequenze, nel gruppo di intervento o nel gruppo di controllo: il primo effettuerà subito il training con il sistema CareToy, mentre il gruppo controllo proseguirà lo studio effettuando la *standard care*.  La randomizzazione del campione nei due gruppi seguirà un rapporto di allocazione pari a 1:1, ossia avrà la stessa probabilità che si ha nel lancio di una moneta.  Lo sperimentatore non parteciperà al processo di randomizzazione che verrà effettuata da terzi non coinvolti nello studio. Lo studio sarà comunque aperto, infatti sia lo sperimentatore che i familiari conosceranno la collocazione del soggetto. Tuttavia il team che valuterà con le scale cliniche il campione, sia al baseline (T0) che in follow up (T1, T2, T3), sarà in cieco (*blind assessor*), in questo modo saranno evitati eventuali bias di valutazione.  In questa fase sarà inoltre effettuato un training genitoriale all'uso del sistema Caretoy.  **- FASE 3: I sessione di intervento**  Prima dell'avvio del trial sia pilota che RCT, il CareToy sarà settato, secondo le esigenze di sviluppo di ciascun bambino. Il sistema sarà quindi consegnato per 4 settimane in casa e ciascun bambino potrà effettuare al domicilio l'intervento individualizzato di promozione dello sviluppo (vedi training) con tele-monitoraggio da parte dello staff riabilitativo. Al contempo il gruppo di controllo proseguirà lo studio con la *standard care*.  **- FASE 4 (T1): Valutazione clinica dopo la sessione di intervento; cross over:**  Tutti i bambini arruolati (sia per lo studio pilota che RCT), dopo la prima sessione di intervento, saranno valutati in modo *blind* con una batteria di scale di sviluppo (vedi misure di outcome) allo scopo di individuare le eventuali modifiche e differenze tra i due gruppi.  Inoltre nell’RCT in preparazione alla fase di intervento successiva (II sessione di intervento) i due gruppi si invertiranno (*cross-over*) pertanto il gruppo che nella fase 3 aveva effettuato l'intervento con Caretoy proseguirà lo studio con la *standard care*, invece il gruppo che durante la fase 3 era stato controllo proseguirà lo studio effettuando il training con il sistema CareToy.  **- FASE 5: II sessione di intervento.**  Come per la fase 3, il sistema Caretoy sarà settato secondo le esigenze di sviluppo di ciascun bambino e verrà consegnato alle famiglie. Il training avrà le medesime caratteristiche dalle fase 3.  **- FASE 6 (T2): Valutazione clinica dopo la II sessione di intervento**  Come nella fase 4, anche in questa fase di studio tutti i bambini arruolati saranno sottoposti alla valutazione clinica con specifiche scale di sviluppo (vedi *scale di valutazione*), i valutatori saranno *blind* pertanto non saranno a conoscenza del gruppo di allocamento del bambino.    **- FASE 7 (T3): follow up; valutazione clinica a 18 mesi di età corretta**.  In questa fase i genitori compileranno dei questionari sullo sviluppo del proprio bambino *(Ages & Stages Questionnaire, Social-Emotional Scale of BSID-III),* e sulla relazione genitore-bambino *(Parenting Stress Index)*. Un sottogruppo di bambini, su consenso dei genitori, eseguiranno anche una valutazione dello sviluppo cognitivo.  Per maggiori dettagli sul protocollo clinico dell’RCT vedi Allegato H.  Per la fase pilota, il timeline sarà uguale a quello delineato per l’RCT ma, non prevedendo la randomizzazione e quindi il cross-over, i bambini effettueranno sempre inizialmente il training sperimentale seguito dalla fase di standard care. Inoltre, nella fase di intervento con il CareToy si prevede l’affiancamento del genitore nell’uso del sistema a casa da parte di un operatore clinico (non coinvolto nella fase valutativa) e/o tecnico in modo da fornire un’assistenza specifica nella risoluzione delle problematiche tecniche.  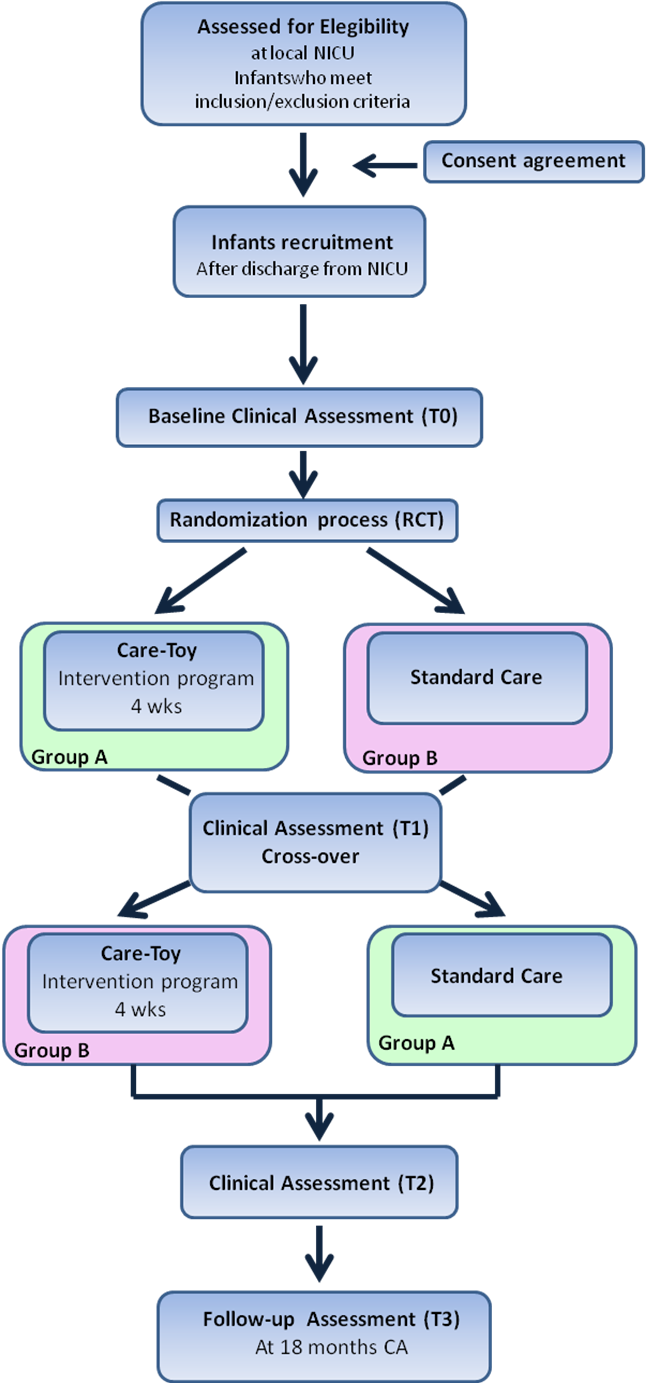  Figura 1: disegno dello studio | |
| Razionale dello studio | | Il tasso delle nascite pretermine in Europa varia dal 5,5% all’11,4%, con una media del 7,1% di tutti i nati vivi. Tale popolazione, rispetto ai bambini nati a termine, presenta a lungo termine una maggiore incidenza di disabilità permanente, in particolare circa il 15% dei bambini nati pretermine riceve una diagnosi di paralisi cerebrale infantile e circa il 50% mostra difficoltà cognitive, motorie o comportamentali (Hack M; 2002). Negli ultimi anni, allo scopo di migliorare l'outcome di sviluppo, sono stati proposti e sperimentati differenti programmi di intervento precoce mirati al bambino, alla sua famiglia ed all'ambiente che lo circonda.  In particolare, con intervento precoce si definiscono tutti quei “programmi di intervento multidisciplinari, attuati precocemente ossia dalla nascita ai 5 anni di vita che hanno l’obiettivo di promuovere la salute dei bambini ed il loro benessere, migliorare le competenze emergenti, ridurre al minimo il ritardo dello sviluppo, e le disabilità già esistenti o emergenti, prevenire la perdita di competenze funzionali, e promuovere la genitorialità adattiva e il funzionamento complessivo della famiglia” in bambini che presentano o che sono a rischio di disordini dello sviluppo (Blauw-Hospers and Hadders-Algra, 2005). Questi programmi possono essere orientati principalmente sullo sviluppo motorio, cognitivo e socio-emozionale del bambino e/o sull’ interazione genitori-bambino (Splitte, 2007).  Numerose revisioni sistematiche sui programmi di intervento precoce hanno evidenziato gli effetti positivi a breve termine che questo tipo di approccio ha sullo sviluppo psico-motorio; tuttavia l' efficacia nel lungo termine risulta non ancora pienamente provata (Orton 2009).  Recenti pubblicazioni scientifiche suggeriscono di includere nei programmi di intervento precoce i principi del Goal Directed Training (GDT) (Siegert, 2004; Löwing K 2010) e dell’ Enriched Home Environment (EHE) (Nithianantharajah, 2006; Guzzetta, 2009) poichè si sono mostrati in grado di potenziare, specie durante le precoci fasi di sviluppo, la plasticità cerebrale, sia nell'uomo che nell' animale (Guzzetta et al 2009; Als et al. 2004).  In breve il GDT si propone, sollecitando l'interesse e l'attenzione del bambino, di fornirgli degli obiettivi il cui raggiungimento prevede un’ attività motoria volontaria. L'EHE è invece un ambiente dotato di caratteristiche atte a stimolare l'interazione sociale e le attività cognitive e motorie del bambino.  Da queste premesse ed ipotesi scientifiche è stato ideato il progetto *Caretoy* che si propone lo sviluppo di un programma di intervento precoce e di promozione dello sviluppo. | |
| Farmaco/i o dispositivo in studio | | Il CareToy H (allegati E1, E2) è un box bio-meccatronico e multisensoriale el cui componenti principali sono:   - 2 pareti multisensoriali dotate di 3 luci grandi, 6 luci più piccole, 4 bottoni e di 3 attacchi per i giochi, - un arco blu con 12 led arancioni per stimolare l'inseguimento visivo e 3 attacchi per i giochi, - 4 giochi biomeccatronici, di forma e dimensione differenti, dotati di dispositivi in grado di rilevare la pressione e la forza utilizzata durante l’afferramento e la manipolazione, - una seduta contenitiva in grado di fornire informazioni sul controllo del tronco in posizione seduta, - un tappetino dotato di sensori di pressione in grado di rilevare la distribuzione del peso del corpo del bambino sul piano di appoggio, - 3 sensori inerziali (chiamati IMUs), che indossati dal bambino durante le sessioni di gioco, sono in grado di rilevare i suoi movimenti all'interno del sistema e consentono una ricostruzione grafica e quantitativa dell’ attività motoria durante il gioco. Due sensori, inseriti all’interno di braccialetti di silicone verranno posti a livello dei polsi del bambino ed un terzo sensore inserito all’interno di una fascia siliconica verra posto sul suo torace. - un monitor, alloggiato in una parete del box, nel quale saranno presentate animazioni finalizzate alla promozione dell'attenzione e dell'inseguimento visivo, - 4 telecamere integrate nella struttura del box, - un sistema di "comunicazione", che sfruttando una connessione internet, consente al CareToy di inviare i dati acquisiti durante la sessione di gioco ad un server collegato direttamente al Centro di Riabilitazione.   Tutti i componenti del sistema sono tra loro integrati ed in grado di rilevare, registrare e quantificare le attività senso-motorie del bambino e fornire dati sul controllo posturale, la funzione manuale, l'attenzione e l'inseguimento visivo e consentono una analisi motoria della attività del bambino durante la sessione di gioco.  Il sistema CareToy-H può essere programmato per proporre al bambino attività ludiche personalizzate (denominate scenari) finalizzate alla promozione dello sviluppo.  Il CareToy Clinical (*CareToy-C*) è un sottosistema ingegnerizzato utilizzato solo presso il centro clinico di riferimento, messo a punto per la valutazione quantitativa del grasping e dell'attenzione visiva nei diversi tempi dello studio (vedi misure di outcome). In particolare, la piattaforma CareToy C è composta da un’unita visione e un’unità toy.  L'unità visione è composta da 5 monitor sui quali vengono presentati degli stimoli visivi, un’ eye-tracker per il monitoraggio dei movimenti oculari e una seduta per posizionare il bambino. Gli schermi sono inseriti in una struttura meccanica di sostegno in modo tale che il bambino sia attratto solo dalle immagini proposte dagli schermi. L'eye-tracker scelto è un sistema SmartEye a 6 telecamere (60Hz).  L’unità toy è un kit di giocattoli sensorizzati (presenti anche nel CareToy H) in grado di misurare le forze di presa esercitate dal bambino durante i compiti di manipolazione mediante dei sensori di forza e pressione posti al loro interno.  In accordo alla definizione di dispositivo medico, mentre il CareToy C è composto da una serie di componenti marcati CE ed utilizzato solo in ambito di ricerca e in strutture specializzate sotto la supervisione di uno staff clinico ed ingegneristico dedicato, il CareToy H è composto principalmente da nuove componenti costruite dai vari partner ingegneristici in accordo alle specifiche fornite dai centri clinici. Tale dispositivo si configura come un dispositivo medico di classe 2 A non dotato di marchio CE, trattandosi di apparecchiatura sperimentale per uso sull’uomo destinata alla ricerca, disegnato secondo le norme fondamentali per coprire i requisiti essenziali di sicurezza (EN 60601-1, 3a ed e EN 60601-1-11) e gli aspetti di compatibilità elettromagnetica (EN 60601-1-2 e EN ETSI 301 489-1). Le prove sperimentali di conformità e l’allestimento della documentazione sono in corso in quanto sono state delegate ad una ditta esterna (Elettra) (vedi allegato E3).  La richiesta di utilizzo del dispositivo il cui inoltro al Ministero della Salute (Direzione Generale farmaci e dispositivi medici Ufficio VI/sperimentazione clinica dei dispositivi medici) è previsto per ogni specifico protocollo, verrà inoltrata appena ottenuta tale documentazione dalla ditta esterna e verrà inviata entro e non oltre il mese di Settembre p.v. | |
| Sinossi (in Italiano) | | Il razionale scientifico del progetto si base sull'evidenza che i programmi di intervento precoce hanno dimostrato efficacia nel migliorare l'outcome di sviluppo di bambini a rischio di disabilità.  Il progetto CareToy propone un intervento precoce domiciliare in bambini pretermine e si propone di valutare l'efficacia dell'intervento precoce con CareToy Home (training sperimentale), rispetto la standard care, sulla promozione in primo luogo dello sviluppo cognitivo ed in secondo luogo dello sviluppo motorio e visivo.  Il CareToy Home è ideato come un sistema modulare integrato attraverso il quale è possibile proporre al bambino sessioni di "gioco" individualizzate e personalizzate finalizzate alla promozione dello sviluppo psico-motorio e visivo. Il sistema è al contempo in grado di rilevare, registrare e quantificare dati sul controllo posturale, la funzione manuale, l'attenzione e l'inseguimento visivo fornendo delle misure quantitative; inoltre, sfruttando la tele-riabilitazione, ne consente l'utilizzo in contesto domiciliare e nel momento di maggiore disponibilità del bambino all'intervento.  Lo studio è un trial clinico sperimentale randomizzato (RCT) di tipo multicentrico e con disegno cross-over [figura 1] nel quale sono coinvolti due centri clinici: la Fondazione IRCCS Stella Maris, in Italia e l'Helen Elsass Center in Danimarca.  Lo studio è disegnato con cross-over per dare l'opportunità, a tutti i bambini arruolati di effettuare un training con CareToy Home.  Il campione sarà costituito da bambini nati pretermine, reclutati dopo la dimissione dalla U.O. di Neonatologia, previa presa in visione del modulo esplicativo del progetto e firma del consenso informato.  I criteri di inclusione indicano come eleggibili i bambini con una età gestazionale compresa tra 28+0 settimane di gestazione e 32+6 settimane di gestazione, in assenza di danno cerebrale (malformazioni, IVH >1 grado, PVL), epilessia o altre forme convulsive note, deficit sensoriali severi (cecità, sordità), scarsa crescita per l'età gestazionale o malformazioni non neurologiche severe (vedi criteri di inclusione/esclusione allo studio). La numerosità del campione è stata calcolata sulla misura di outcome primaria; il campione verrà per metà reclutato in Italia e per metà reclutato in Danimarca.  Al reclutamento saranno acquisiti e registrati su apposito database i dati relativi alla storia pre-perinatale del bambino e alcune informazioni sui familiari. A tutela della privacy a ciascun bambino sarà assegnato un codice numerico, pertanto il database non conterà nessun dato demografico e l'accesso sarà protetto tramite password e limitato al solo personale coinvolto nello studio.  I bambini arruolati saranno allocati, in modo random, con rapporto 1:1, nel gruppo sperimentale o nel gruppo di controllo; tuttavia prevedendo lo studio un cross over, i due gruppi verranno scambiati subito dopo la prima fase così che il gruppo sperimentale diventerà controllo ed il gruppo controllo effettuerà il training sperimentale.  Lo sperimentatore non parteciperà al processo di randomizzazione, ma lo studio sarà aperto, tuttavia per ridurre i bias, il team clinico che valuterà il campione, sia al baseline che in follow up (T0, T1, T2, T3), sarà in cieco (blind assessor).  Dopo il reclutamento ai genitori sarà chiesto di compilare, a cadenza mensile, un questionario sulle abilità grosso-motorie del bambino allo scopo di individuare, per ciascun bambino, il momento più opportuno per l'avvio della fase sperimentale (vedi misure di outcome Ages & Stages Questionnaire). Prima dell'avvio del training sperimentale il sistema CareToy sarà programmato, in accordo con le esigenze e le potenzialità di sviluppo di ciascun bambino, fornendo un intervento personalizzato. La durata totale del training è di 4 settimane, durante le quali saranno proposte sessioni di gioco quotidiane (5 giorni a settimana) della durata di 30-40 minuti/die.  Durante il periodo di studio il campione sarà valutato (sul piano cognitivo, motorio e visivo) attraverso scale cliniche standardizzate e con un sistema ingegnerizzato del CareToy chiamato, CareToy Clinico (C), messo a punto per la valutazione quantitativa del grasping e dell'attenzione visiva. Le valutazioni cliniche verranno effettuate in 4 tempi: nella settimana precedente all'avvio del training sperimentale/standard care (T0), nella settimana successiva al primo periodo di training sperimentale/standard care (T1), nella settimana successiva al secondo periodo di training standard/sperimentale; dopo il cross-over (T2) ed a 18 mesi di età corretta (T3); (vedi tabella 1). | |
|  |  | Osservazioni: | |
| Protocollo | *Obiettivi e endpoints* | **Obiettivi**  L’obiettivo del progetto è quello di promuovere lo sviluppo psicomotorio di bambini nati pretermine attraverso un programma individualizzato di intervento precoce.  L’obiettivo principale è quello di promuovere i processi di sviluppo cognitivo ed in particolare l’ attenzione, la relazione oggettuale, la memoria, l’abilità di problem solving e le competenze di esplorazione e di manipolazione dell'oggetto.  Come obiettivo secondario lo studio si propone di valutare l'efficacia del programma di intervento sullo sviluppo:   - Visivo: in particolare dell’ acuità e dell’attenzione visiva - Motorio: sia in termini quantitativi di competenze posturo-motorie e di abilità manuali (in particolare del grasping e della forza di afferramento) che qualitativi quali la valutazione del repertorio motorio e delle abilità di selezione adattiva.   Infine si propone di sperimentare la tele-riabilitazione in una popolazione di bambini nati pretermine.  **ENDPOINT**  L’endpoint primario è il maggior cambiamento a breve termine nella misura di outcome primario nei bambini sottoposti al training con CareToy rispetto alla standard care. Secondariamente, ci si aspettano dei cambiamenti a breve termine anche nell’ambito dello sviluppo visivo e motorio. Inoltre, nel follow-up a lungo termine (T3) ci si aspetta un outcome positivo nello sviluppo psicomotorio e cognitivo dei bambini arruolati nello studio. | |
|  | *Tipologia dei pazienti* | ll campione di studio sarà costituito da bambini nati pretermine che soddisfino i criteri di inclusione ed esclusione allo studio (vedi oltre) che verranno reclutati sia in Italia che in Danimarca.  Il reclutamento dei bambini in Italia sarà effettuato prevalentemente da bambini dimessi dall'U.O. di Neonatologia dell'AO Pisana. | |
|  | *Criteri di inclusione* | Criteri di Inclusione:   - Età gestazionale ≥ 28+0 settimane di gestazione e 32+6 settimane di gestazione - Età corretta a T0 tra 2 e 9 mesi e raggiungimento del punteggio limite, in rapporto all'età corretta, nell'area relativa alle abilità grosso-motorie del questionario Ages & Stages Questionnaire (ASQ-3); in dettaglio: - punteggio ≥ 25 all' ASQ-3, 2 mesi (comprende la fascia di età tra 1 mese e 2 mesi e 30 giorni) - punteggio ≥ 10 all' ASQ-3, 4 mesi (comprende la fascia di età tra 3 mesi e 4 mesi e 30 giorni) - punteggio ≥ 5 ed ˂ 50 all' ASQ-3, 6 mesi (comprende la fascia di età tra 5 mesi e 6 mesi e 30 giorni) - punteggio ≥ 10 ed ˂ 30 all’ ASQ-3, 8 mesi (comprende la fascia di età tra 7 mesi e 8 mesi e 30 giorni) | |
|  | *Criteri di esclusione* | Criteri di esclusione   - Età gestazionale < 28 settimane o ≥ 33 settimane - Piccolo per età gestazionale (SGA) - Presenza di lesioni cerebrali (malformazioni congenite, esiti ipossico-ischemici, emorragia intraventricolare > 1 grado o leucomalacia periventricolare di ogni grado) - Epilessia o altre forme convulsive note - Deficit sensoriali severi (cecità, sordità) - Altre malformazioni non neurologiche severe - Arruolamento simultaneo in altro studio sperimentale con finalità riabilitative | |
|  | *Criteri di uscita* | - ritiro del consenso informato  - insorgenza di accertata epilessia o di fotosensibilità  - peggioramento del quadro clinico | |
|  | *Valutazione di risposta e sicurezza del trattamento (visite, follow-up, esami)* | Come precedentemente descritto il campione effettuerà 4 valutazioni cliniche a T0, T1, T2, T3. Tali valutazioni prevedono l'impiego di strumenti valutativi standardizzati e specifici per l'età evolutiva (vedi tabella 1), tali test sono in grado di fornire indici di sviluppo quantitativi e qualitativi.  Le valutazioni del gruppo di bambini reclutati in Italia si svolgeranno presso l'IRCCS Fondazione Stella Maris, i bambini reclutati in Danimarca saranno invece valutati presso l'HEC.  Gli operatori coinvolti nelle valutazioni cliniche presso i due centri clinici (HEC e FMS) hanno effettuato dei training per ottenere una concordanza nello scoring pari al 100%.  **Misura di outcome primaria:**  In accordo con l'obiettivo principale dello studio, la misura di outcome primaria è la   - Bayley III; *Cognitive subscale (T0, T1, T2, T3*)*   Le BSID-III sono scale standardizzate per l'assessment dello sviluppo funzionale di bambini da 1 a 42 mesi di età. Queste scale permettono di identificare i bambini con ritardo nello sviluppo e sono impiegate e validate anche per bambini nati pretermine.  In particolare la sottoscala Bayley III cognitiva (*Cognitive subscale)* è in grado di valutare lo sviluppo senso-motorio, l'esplorazione e la manipolazione, la formazione di concetti, la memoria e il problem-solving ed altri aspetti del processo cognitivo.  **Misure di outcome secondarie:**  Allo scopo di valutare l'efficacia del CareToy sulla promozione dello sviluppo posturale, della funzione manuale e dello sviluppo visivo (vedi obiettivi) sono state scelte le seguenti scale valutative:  Valutazione Motoria:   - *Alberta Infant Motor Scale (AIMS)*; *(T0, T1 e T2)*   É uno strumento validato per la valutazione dello sviluppo motorio, in termini di competenze posturo-motorie, per bambini fino ai 18 mesi di età.  *- Infant Motor Profile (IMP)*; *(T0, T1 e T2)*  L'IMP valuta il comportamento motorio spontaneo del bambino in un’ età compresa tra i 3 e i 18 mesi, o più precisamente fino a quando il bambino ha acquisito una buona esperienza di cammino autonomo. In breve lo strumento valuta non soltanto le prestazioni motorie in termini quantitativi, ma anche qualitativi quali la variabilità e ka fluidità del movimento e l’ adattabilità delle strategie motorie. E' uno strumento validato sia nei bambini nati a termine che pretermine.  - *CareToy C: valutazione del grasping (T0, T1 e T2)*  La valutazione del grasping tramite il CareToy C prevede l'impiego di giochi bio-meccatronici dotati rispettivamente di sensore di pressione che consentirà di quantificare la forza di grasping e il numero di prese (vedi Descrizione).    Valutazione Funzionalità Visiva:   - *Teller acuity Cards (T0, T1 e T2)*   Il test consente la valutazione dell’ acuità visiva anche in bambini con età inferiore all'anno di vita. Al bambino viene mostrata una tavola (card) che presenta da un lato un target visivo costituito da strisce bianco/nere di diversa larghezza e dall'altro uno stimolo neutro (sfondo grigio). Il test valuta la capacità del bambino di dirigere lo sguardo verso il target visivo. La stima dell'acuità visiva si basa sulla più fine larghezza di strisce che il bambino fissa e quindi preferisce all'area neutra. Il test si basa quindi sul principio del "preferential looking", gli indicatori di risposta sono su base comportamentale spontanea es. orientamento dello sguardo o del capo verso lo stimolo. Il test è di elevata attendibilità, è versatile e richiede brevi tempi di esecuzione.  - *CareToy C: valutazione visiva (T0, T1 e T2)*  La valutazione visiva tramite il CareToy C prevede l'impiego di un sistema di eye tracker con la finalità di misurare lo spostamento del gaze e la risposta visiva attentiva secondaria alla presentazione di uno stimolo visivo (vedi Descrizione).  Questionari compilati dai genitori:   - *Ages & Stages Questionnaire*; *(T0, T3):*   Il questionario è in grado di valutare lo sviluppo psicomotorio di bambini da un 1 a 66 mesi di età. Per il nostro studio dapprima l’area delle abilità grosso-motorie sarà utilizzata con lo scopo di individuare, in ciascun bambino, il momento più opportuno per l'avvio della fase sperimentale attraverso il raggiungimento di uno score minimo (vedi criteri di inclusione). Il questionario nella sua totalità verrà compilato dai genitori a T0 e sarà inoltre riproposto a T3 per monitorare lo sviluppo dei bambino a lungo termine.   - *Bayley III*; *Social-Emotional subscale* *(T0, T1, T2 and T3):*   E' una sottoscala della Bayley III che consente di identificare eventuali difficoltà nel processamento delle emozioni e di ottenere un indicatore del livello di sviluppo sociale ed emotivo del bambino. In particolare consente di valutare la padronanza che il bambino ha della propria funzionalità emotiva, dei bisogni comunicativi, della capacità di relazionarsi con gli altri, di utilizzare le emozioni in modo interattivo e finalizzato e l'uso dei segnali emotivi per risolvere i problemi.   - *Parenting Stress Index (PSI) (T0, T1, T2 and T3)*   Lo strumento esplora le dimensioni emotive e comportamentali dei bambini e valuta il rischio per il genitore di vivere come disfunzionale il proprio ruolo.  Tabella 1   \|  \| **T0** \| **T1** \| **T2** \| **T3** \| \| --- \| --- \| --- \| --- \| --- \| \| **Misura di outcome primaria** \|  \|  \|  \|  \| \| Bayley III Cognitive Subscale \| ✓ \| ✓ \| ✓ \| ✓^*^ \| \| **Misure di outcome secondary** \|  \|  \|  \|  \| \| AIMS \| ✓ \| ✓ \| ✓ \|  \| \| IMP \| ✓ \| ✓ \| ✓ \|  \| \| Teller Acuity Cards \| ✓ \| ✓ \| ✓ \|  \| \| CareToy C measures \| ✓ \| ✓ \| ✓ \|  \| \| **Questionari** \|  \|  \|  \|  \| \| Parenting Stress Index \| ✓ \| ✓ \| ✓ \| ✓ \| \| Ages & Stages Questionnaire \| ✓ \|  \|  \| ✓ \| \| Bayley III Social-Emotional Subscale \| ✓ \|  \|  \| ✓ \|  Valutazione della compliance al training:Durante il training sperimentale, con cadenza settimanale, le sessioni di intervento saranno esaminate *"in remoto"* dallo staff clinico allo scopo di:- valutare l'evoluzione del bambino- adattare il programma di training alle esigenze di sviluppo del bambino. - valutare la compliance del bambino al training  Inoltre, tramite una breve intervista on line, sarà chiesto ai genitori di fornire giornalmente e settimanalmente informazioni sulla partecipazione e la compliance del bambino al training. | |
|  | *Analisi statistica (numerosità campionaria e metodo)* | Sample size  La dimensione del campione è stata scelta sulla base dei cambiamenti previsti nella misura di outcome primaria (*Bayley III, Cognitive subscale, vedi misure di outcome*).  In base ai risultati emersi dalla revisione della letteratura, in uno studio di Mazurek Melnyk et al 2001, in cui è stata valutata l'efficacia di programma di intervento mirato sul genitore (COPE) sullo sviluppo cognitivo nei bambini pretermine, la risposta al cambiamento alla scala cognitiva BSID III è risultata molto elevata con un *effect size* a 3 e 6 mesi di età corretta compresa tra 0,60 e 0,72.  Secondo un calcolo statistico, considerando un livello *alfa* di *0.05* con una potenza dell'80% ed un effetto di almeno 0.6, per il nostro studio avremmo bisogno di 45 sessioni "sperimentali" e 45 sessioni "controllo", a cui sarà necessario aggiungere un 20% di sessioni per coprire la possibilità di *drop-outs*. In totale sarà quindi necessario effettuare 108 sessioni (sperimentali e controllo). Prevedendo lo studio un *cross-over* tra il gruppo sperimentale e controllo i soggetti reclutati saranno assegnati, in tempi diversi, ad entrambi i bracci dello studio (controllo e sperimentale), per cui per il nostro intento saranno necessari 54 bambini.  Il campione verrà per metà (27 soggetti) reclutato in Italia e per metà (27 soggetti) reclutato in Danimarca.  I gemelli saranno assegnati allo stesso gruppo nei medesimi tempi allo scopo di facilitare i genitori nella gestione del Sistema Caretoy per entrambi i bambini allo stesso tempo.  Per maggiori dettagli sul protocollo clinico vedi Allegato H. | |
|  | *Case report form (CRF)* | Allegato A e A1 | |
| Testo informativo per i genitori del bambino | | Allegato B | |
| Dichiarazione di consenso del genitore | | Allegato C | |
| Lettera al medico di medicina generale | | Allegato D | |
| Clinical investigator’s brochure (CIB) o scheda tecnica del dispositivo | | Allegato E1, E2 ed E3 | |

| Altre osservazioni |  |
| --- | --- |
| Deliberazione | Approvato - condizioni/raccomandazioni – Rinviato – Respinto |
