# Supplementary material for: A randomized clinical trial in preterm infants on the effects of a home-based early intervention with the 'CareToy System'
Source: PLoS One. 2017 Mar 22;12(3):e0173521. doi: 10.1371/journal.pone.0173521 (PMC5362053; doi:10.1371/journal.pone.0173521)
Supplement: S2 Text — (DOC) [file pone.0173521.s003.doc]

**SUMMARY STATEMENT OF PROTOCOL OF CLINICAL STUDY**

| identifying elements | | | |
| --- | --- | --- | --- |
| Title of the study | “CareToy: a new intelligent modular system based on biomechatronic games for home rehabilitation of children” | | |
| Version and date of the protocol | Version of the Code of the Research: CareToy ICT-STREP proposal number 287932 | | |
| Documents  (version and  date) | Letter of presentation (version 1.0 of 20 June 2013)  Annex A: Case Report Form (IT)  Annex A1: Evaluation protocol (IT)  Annex B: Information prospectus for the patient (IT)  Annex C: Consent Form for the patient (IT)  Annex D: Consent Form for the patient (IT)  Annex E1: Synopsis on the CareToy H Specifications (IT)  Annex E2: Deliverable MS-2 (Technical description of CareToy components) (EN)  Annex E3: Trials for device (quote of Elettra) (IT)  Annex F2: Insurance Policy  Annex F3: Quote Insurance  Annex G: CareToy Consensus Agreement (EN)  Annex H: Ethical issues (Deliverable 7.3) (EN)  Annex I: May 2013 study protocol (Deliverable 7.1) (EN)  Annex L: full research project approved by the European Union (EN)  Annex M: Curriculum investigator (IT) | | |
| Codes | *OSC EUDRACT:*  NA | *Promoter of the trial:* | *FSM:*  ………./2013 |

| Administrative Info | | | |
| --- | --- | --- | --- |
| Experimenter main action, name and registered office | | | Principal investigator in Italy:  Prof. Giovanni Cioni  IRCCS Fondazione Stella Maris  Viale del Tirreno 341/ ABC  56128 Calambrone (Pisa) – Italia  Another European investigator center (Denmark)  Helene Elsass Center  Holmegårdsvej 28  2920 Charlottenlund  Copenhagen – Denmark  Coordinating center:  The BioRobotics Institute - Scuola Superiore Sant'Anna Polo Sant'Anna Valdera  Viale Rinaldo Piaggio 34  56025 Pontedera (Pisa) – Italia |
| Coordinating center: | | | Prof. Giovanni Cioni  IRCCS Fondazione Stella Maris  Viale del Tirreno 341/ ABC  56128 Calambrone (Pisa) – Italia |
| Letter of investigator | | | See letter |
| List of participating centers | | | - BioRobotics Institute – Scuola Superiore Sant’Anna, Italia - IRCCS Fondazione Stella Maris, Italia - STMicroelectronics SRL, Italia - Univerza V Ljubljani, Slovenia - Universitaet Hamburg, Germany - Fonden for Helen Elsass Center, Denmark - Marketing Research & Development SPA, Italia |
| Start-end date of the study | | | November 2013 – April 2015 |
| Insurance | | | The IRCCS Fondazione Stella Maris has before proceeded to contact the broker getting the specific prices (see Appendices F1, F2 and F3) for the CareToy project that will be signed as soon as the final prototypes will be produced and the recruitment will start.  The IRCCS Fondazione Stella Maris has taken out the insurance policy from 1/11/2013 to 31/10/2014 (Annex 4) |
| Economic Convention | | | The CareToy project is funded by the European Community. |
| Data ownership | | | As will be specified later (see Recruitment), to protect the privacy and anonymity, at the time of recruiting IRCCS Fondazione Stella Maris will allocate to each child a numeric code which will be stored in separate form so that the database does not contain demographic and clinical data. Access to these data will be limited to the Institute's staff directly involved in the study and all the data will be processed anonymously.  The project data treated in a general form will be a property of all members of the CareToy consortium as stated in the Consensus Agreement signed by all parties (see Annex G). |
| Drug supply | | | In the study design it is not allowed the use of drugs |
| Scientific DATA of the study | | | |
| Phases and Design | | The experimental study will be performed in two phases. At the beginning, a pilot phase will be carried out in order to test, on a small group of children (max 10) the study protocol aimed at improve the functioning of the CareToy H system from a technical point of view (in the software of the games and on the protocols of the data analysis transmission). The pilot phase involves first testing of Caretoy (experimental training) for 4 weeks and then the control period of the same duration.  Then, a multicenter and cross-over [Figure 1] Randomized Clinical Trial (RCT) will be carried out in order to compare the effects of the CareToy Home (H system) intervention program(experimental training) with standard care.  Enrolment and clinical trials for both phases (pilot and RCT) will take place in paralle in the two involved clinical centers, in Italy at the Stella Maris Foundation and in Denmark at Helen Elsass Center.  The study RCT has been designed with cross-over to give the opportunity to all children enrolled to make a training with CareToy H system.  The total duration of the project is 3 years, of which one year will be dedicated to clinical trials.  Experimental Training: The training with CareToy provides daily use (30-40 minutes per day) for 5 days per week for 4 weeks consecutive of CareToy H System, a bio-mechatronic and multisensory box, designed as a modular smart system which employs innovative technological strategies that detect, record and quantify the activities of the baby inside the box by providing data on the visual and attentive postural-motor and the manual skills, (for details see medical device). Inside the CareToy H personalized and individualize psycho-motor activities are proposed aimed at promoting psychomotor development. The daily "game" activities carried out within the CareToy H system could be also divided in 3 sessions of approximately 10 minutes or in more sections according to the availability of the baby and his parents. The system, in fact, exploiting the use of tele-rehabilitation, allows to make the training at home, but under monitoring by the rehabilitative staff (child psychiatrists and developmental therapists). The child will then use the CareToy H in his own home but the data, acquired during the training session, will be automatically uploaded to a server connected to the Rehabilitation Clinical Center and so the rehabilitation staff may remotely monitor and plan the activities, evalute the progresses and therefore reprogram the new activities to be proposed based on the needs of each child. In the days prior to the delivery of the system at home, the family will be instructed to use the CareToy H through parental training and in the first week of training and, if necessary, in the following weeks, there will ne a clinical and technical assistance at home.  Before and after the period of experimental training / control period children will be evaluated in a exact time (see Timeline) with clinical scales and an engineered subsystem of CareToy called CareToy Clinical (C) developed for quantitative assessment of grasping and of ' visual attention (see outcome measures and description of device).  **TIMELINE:**  TIMELINE:  The sample, during the study period RCT, will be evaluated in 4 different times: T0, T1, T2, T3 (see table 1). The evaluations will take place in Italy at the IRCCS Stella Maris, Calambrone and will involve children and parents for 2 days.    - T0: in the week before the start of the training  - T1: in the week following the first period of experimental training / standard care  - T2: the week after the second period of standard / experimental training (cross-over)  - T3: 18 months after the end of training.    Table 1 shows the times of use of rating scales and questionnaires.    The clinical phase of the study can be broadly divided into:  A) STEP 1 (pre-T0):  1) Identification of the recruited cases  According to the inclusion / exclusion criteria, the eligibility of the sample will be evaluated by the team from the Department of Neonatology Unit of Azione Ospedaliera Pisana, Santa Chiara Hospital.  At the moment of discharge from the hospital, the parents of eligible children will be informed of the general aims of the project and if they will express interest they will be contacted by the team of IRCCS Fondazione Stella Maris that will expose in detail the research project and, if the family will give verbal consent to participate in the study, they will sign the written informed consent.    2) Recruitment  Only after having obtained from the parents the written consent to participation in the study, the child will be recruited.  During recruitment, the CRF (see Annex) will be filled in through parental interview and examination of clinical documents, and the clinical data (sex, gestational age), the data of prenatal history (mother primipara/multipara, high-risk pregnancy e.g. for advanced maternal age, gestational diabetes, hypertension ...), perinatal (type of delivery, birth weight, APGAR score, Details of the clinical perinatal history) and the results of screening and / or any further instrumental examinations (eg. brain ultrasound) will be acquired and registered, in a special database. In addition it will be done ​​a general and neurological clinical examination of the child and if at the time of recruitment the child will have a correct age less than 20 weeks, an assessment of spontaneous motor activity with video recording according to Prechtl method's (Qualitative Assessment of General Movements ) will be also carried out.  To protect the privacy and anonymity, a numeric code which will be stored in separate form will be initially assigned to each child so that the database will not contain demographic and clinical data. Access to these data will be limited only to local staff directly involved in the study and all the data will be processed anonymously.  After the recruitment, the parents will be asked to fill in monthly a questionnaire (Ages & Stages Questionnaire) on child development in order to identify, on the basis of gross motor skills acquired during the first months of life, when the child will reach the minimum score (see Inclusion criteria and outcome measures) for each child, the time when he will be able to entry in the experimental stage.  **- FASE 2 (T0): Assessment at baseline and randomization**  The evaluation will be performed at baseline when the child will have a correct age between 2 and 9 months and will have acquired gross- fine motor skills in accordance with the inclusion criteria (score Ages & Stages Questionnaire, see inclusion criteria).  During the evaluation at baseline the use of specific development scales and of questionnaires filled in by the parents (for details see "outcome measures") is provided.  After evaluation, the sample enrolled in the RCT will be randomly allocated by a computer generator sequences, to the intervention or the control group: the first group will carry out immediately the training with CareToy system, while the control group will continue the study by performing the standard care.  The randomization of the sample in the two groups will follow an equal allocation ratio 1: 1, with the same probability that there is in the toss of a coin.  The investigator do not participate in the randomization process that will be carried out by third parties not involved in the study. The study will be open, in fact, both the investigator and the family will know the location of the subject. However the team that will evaluate the children with the clinical scales, both at baseline (T0) and in the follow-up (T1, T2, T3), will be blinded (blind assessor). In this way any assessment bias will be avoided.    In this phase a parental training for the use of Caretoy system we will performed.  **- FASE 3: I intervention session**  Before the trial, both pilot and RCT, the CareToy will be set according to the needs of each child development. The system will then be delivered for the four weeks to the house and each child will perform the individualized intervention aimed to promote his development (see training) with the tele-monitoring by the rehabilitation staff. At the same time, the control group will continue the study with the standard care.  **- FASE 4 (T1): Clinical assessment after the intervention session; cross over:**  After the first intervention session, all enrolled children (both for the pilot and RCT study), will be blindly evaluated with a battery of developmental scales (see outcome measures) in order to identify any changes and differences between the two groups.  In addition, in the RCT, the two groups will be inverted for the next stage of intervention (II intervention session) (cross-over). Therefore, the group that in step 3 had carried out with the Caretoy intervention followed the study with standard care, while the group that during phase 3 was allocated in the control group, followed the study with CareToy training.  **- FASE 5: II session of intervention.**  As for Phase 3, the Caretoy system will be set according to the development needs of each child and it will be delivered to the families. The training will have the same characteristics of Phase 3.  **- FASE 6 (T2): Clinical evaluation after the II session of intervention**  As in phase 4, also in this phase of the study all enrolled children will be evaluated with specific developmental scales (see evaluation) by evaluators to the allocation.    **- FASE 7 (T3): follow up; clinical assessment at 18 months of corrected age**.  At this stage the parents will fill in questionnaires on their child's development (Ages & Stages Questionnaire, Social-Emotional Scale of BSID-III), and the parent-child relationship (Parenting Stress Index). A subgroup of children, after specific parental consent , will also perform an evaluation of cognitive development.  For more details on the clinical protocol of RCT see Appendix H.  For the pilot phase, the timeline will be equal to that outlined for the RCT but not providing randomization and thus the cross-over, all the children will initially carry out the experimental phase of training followed by standard care. Moreover, in the phase of intervention with the CareToy it is expected for the parents the assistance at home of a clinical operator (not involved in the evaluation phase) and / or of a technician in order to provide specific assistance in the resolution of technical issues.  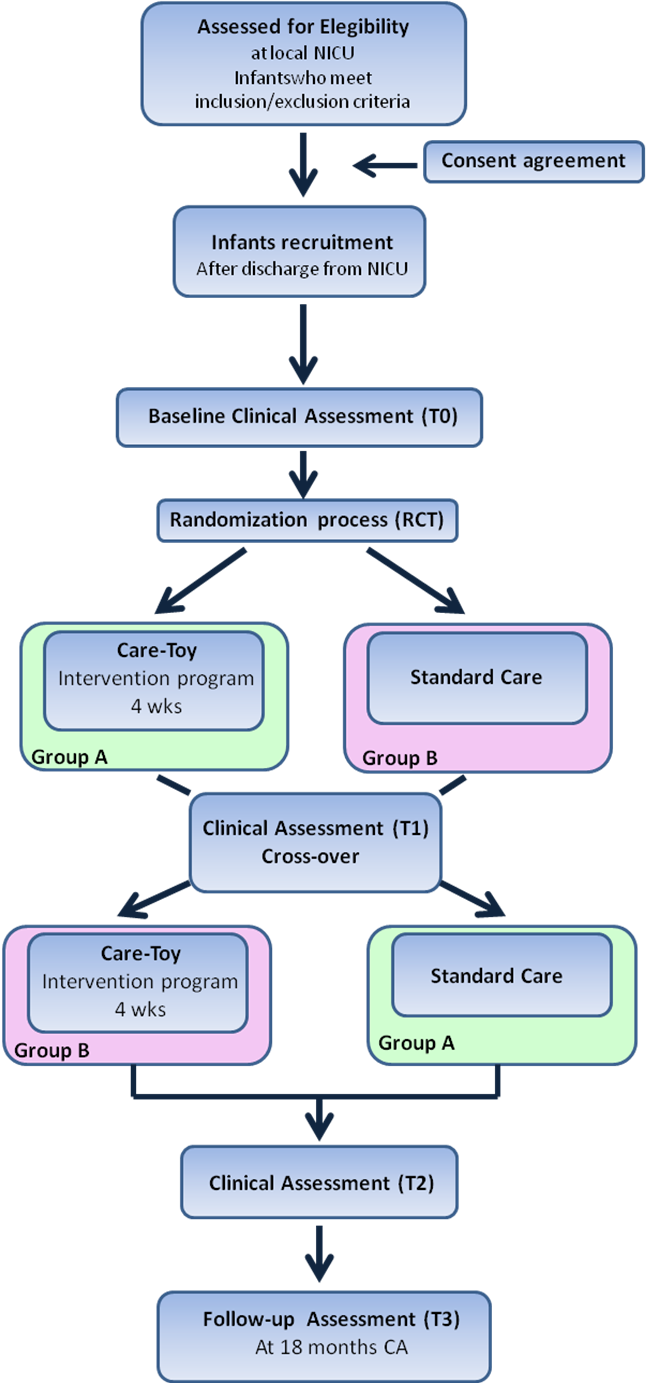  Figura 1: Study design | |
| Rationale of the study | | The rate of preterm births in Europe ranges from 5.5% to 11.4%, with an average of the 7.1% of all live births. This population, compared with children born at term, presents a long-term higher incidence of permanent disability, in particular about 15% of babies born preterm has a diagnosis of cerebral palsy and approximately 50% show cognitive, motor or behavioural disabilities (Hack M, 2002). In recent years, in order to improve the outcome of development, different intervention programs have been proposed and tested with the child, his family and the environment.  In particular, with early intervention are defined all "Multidisciplinary intervention programs, early preformed ie from birth to five years of life that have the goal of promoting children's health and well-being, improving emerging skills, minimize developmental delay and disabilities already existing or emerging, prevent the loss of functional skills, and promote adaptive parenting and overall family functioning " in children who have or are at risk of developmental disorders (Blauw-Hospers and Hadders-Algra, 2005).  These programs can be oriented mainly on motor development, cognitive and socio-emotional child and / or on parent-child interaction (Splitte, 2007).  Several revisions on early intervention programs have highlighted the short-term positive effects that this type of approach has on the psycho-motor development; however, the long-term efficacy is not yet fully proven (Orton 2009).  Recent scientific publications in the early intervention programs suggest to include the principles of Goal Directed Training (GDT) (Siegert, 2004; Lowing K 2010) and Enriched Home Environment (EHE) (Nithianantharajah, 2006; Guz Journal, 2009) because they have shown to be capable of strengthening, especially during the early stages of development, brain plasticity, both in humans and in animal models (Guzzetta et al 2009; Als et al. 2004).  In short the GDT, promoting the interest and attention of the child, aims to provide the the achievement of goals which requires a voluntary motor activity. The EHE is rather an environment equipped with features to encourage social interaction and cognitive and motor activities of the child.  From these premises and scientific hypotheses the Caretoy project is designed with the aim to develop an early intervention program and promote development. | |
| Drug/Medical Device | | The CareToy H (Annexes E1, E2) is a bio-mechatronic and multisensory box and its main components are:   - 2 multisensory walls with three big lights, 6 smaller lights, 4 buttons and 3 points for the sensorized toys, - a blue arch with 12 LED orange to stimulate the visual following and 3 points for sensorized toys, - 4 biomechatronic toys , of different shape and size, equipped with sensors able to detect the pressure and the force used during grasping and manipulation, - a comfortable seat able to provide information on the control of the trunk during the sitting position, - a mat equipped with pressure sensors able to detect the distribution of the child's body weight on the support surface, - 3 inertial sensors (called IMUs) that, worn by the child during the training sessions, are able to detect his movements within the system and to allow a quantitative and graphic reconstruction of motor activity during the training. Two sensors, inserted inside of silicone bracelets, will be placed in the child's wrists level and a third sensor, placed inside a silicone band, will be placed on his chest. - a monitor, placed in a wall of the box, in which animations aimed at promoting attention and visual following will be presented, - 4 cameras integrated in the structure of the box, - a "communications" system that through an Internet connection, allows the CareToy to send the data acquired during the training session to a server connected directly with the Rehabilitation Center.     All system components are integrated and they can detect, record and quantify the child’s sensory-motor activities and provide data on his postural control, manual function, visual attention and following. These data allow the analysis of motor activity of the child during the training session.  The CareToy-H system can be programmed to propose the personalized play activities (called scenarios) aimed at promoting child’s development.    The CareToy Clinical (CareToy-C) is an engineered subsystem used only at the clinical center, developed for quantitative assessment of the grasping and visual attention in different times of the study (see outcome measures.) In particular, the CareToy C platform is composed of a unit vision and a toy unit.  The vision unit is composed of five monitors on which are presented visual stimuli, an eye-tracker for measuring eye movements and a chair where the child is placed. The screens are incorporated into a mechanical support structure in such a way that the child is attracted only by the images proposed by the screens. The eye-tracker used is the SmartEye system with 6 camera (60Hz).  The toy unit is a kit of sensorized toys (also used in CareToy H) able of measuring the grip forces exerted by the child during manipulation tasks by means of the force sensors and pressure places inside them.    In accordance with the definition of medical device, while the CareToy C is composed of a series of CE-marked components and used only in research field and in specialized facilities under the supervision of a clinical and technical staff, the CareToy H consists mainly of new components built by various technical partners in accordance with the specifications provided by clinical sites. The CareToy H is configured as a medical device class 2A without CE mark, since it is an experimental equipment for use on humans research, designed according to the basic rules to cover the essential safety requirements (EN 60601-1, 3rd and EN 60601-1-11) and electromagnetic compatibility aspects (EN 60601-1-2 and ETSI EN 301 489-1). The experimental tests of compliance and preparation of documentation are being as they are delegated to an outside company (Elettra) (see Annex E3).  The request for approval on use of the CareToy H to the Ministry of Health, needed for each specific protocol, will be sent as soon as (no later than September pv) we will obtain the documents by the Elettra company. | |
| Synopsis | | The scientific rationale of the project is based on evidence that early intervention programs seem effective on improving the outcome of children at risk for developmental disabilities.  The project proposes a CareToy home early intervention in preterm children and it aims to evaluate the efficacy of early intervention with CareToy Home (experimental training), compared to standard care, in promoting firstly cognitive development and secondly the motor and visual development.  The CareToy Home is designed as an integrated modular system through which you can bring the child to "play” with individualized and customized activities able to promote the psycho-motor and visual development. At the same time, the system is able to detect, record and quantify data on postural control, manual function, attention and visual following. Moreover, taking advantage of the tele-rehabilitation, it is used in the home environment and when the child is more active  The study is a multicenter randomized experimental clinical trial (RCT) with a cross-over design [Figure 1]. The two clinical centers involved are: IRCCS Fondazione Stella Maris, in Italy and Helen Elsass Center in Denmark.  The study is designed as a cross-over to give the opportunity to deliver the CareToy system to all the children enrolled.  The sample will consist of children born preterm, recruited after discharge from the Unit of Neonatology, after signature of the informed consent.  The inclusion criteria indicate as eligible children with a gestational age between 28 + 0 weeks of gestation and 32 + 6 weeks of gestation, in the absence of brain damage (malformations, IVH> 1 degree, PVL), epilepsy or other seizures, severe sensory impairment (blindness, deafness), poor growth for gestational age, or severe no neurologic abnormalities (see criteria for inclusion / exclusion in the study). The sample size was calculated on the primary outcome measure; the sample will be recruited, half in Italy and half in Denmark.  At the moment of Recruitment, data relating to pre-perinatal history of the child and some information on family members will be recorded in a special database. To protect the privacy a numeric code will be assigned to each child, so that the database will not have any demographic data and its access will be protected by a password and the use limited only to personnel involved in the study.  Enrolled children will be allocated, randomly, with the ratio 1: 1, in the experimental group or the control group. However, for the cross-over design, the two groups will be exchanged immediately after the first stage so that the experimental group will become control and the control group will carry out the experimental training.  The investigator will not participate in the randomization process, but the study will be open. However, to reduce bias, the clinical team that will evaluate the children, will be blind (blind assessor) both at baseline and in follow-up (T0, T1, T2, T3) assessments.  After recruitment, parents will be asked to fill in monthly a questionnaire on the gross motor skills of the child in order to identify, for each child, the most appropriate time for the start of the experimental phase (see outcome measures Ages & stages Questionnaire). Before the experimental CareToy training the system will be programmed in accordance with the needs and the potential development of each child in order to provide a personalized intervention. The total training time is 4 weeks, during which daily rehabilitative sessions will be proposed (5 days per week) with a duration of 30-40 minutes / day.  During the study period, the sample will be evaluated (at the cognitive, motor and visual development) through standardized clinical scales and with an engineered CareToy system called CareToy Clinical (C), developed for the quantitative assessment of the grasping and visual attention. Clinical evaluations will be carried out in 4 stages: in the week before the start of the experimental training / standard care (T0), during the week following the first period of experimental training / standard care (T1), in the week following the second standard period /experimental training, after the cross-over (T2) and at 18 months of corrected age (T3); (see table 1). | |
|  |  | Remarks: | |
| Protocollo | *Objectives and endpoints* | **Objectives**  The goal of the project is the promotion of psychomotor development of children born preterm through an individualized program of early intervention.  The main objective is the promotion of the cognitive development and in particular the attention, the object relationship, memory, problem solving abilities and exploration skills and object manipulation.  As a secondary objective the study aims to evaluate the effectiveness of the intervention program on the development:  - Visual: in particular of visual acuity and attention  - Motor: both in terms of quantity of postural-motor skills and manual skills (particularly the grasping and grasping force) and of qualitative aspects of motor repertoire and adaptive selection skills.  Finally it proposes to test the tele - rehabilitation in a population of children born preterm.  **ENDPOINT**  The primary endpoint is the short-term change in the primary outcome measure more in children that have carried out the training with CareToy compared to standard care. Secondly, we expect short-term changes even within the visual and motor development. Moreover, in the long-term follow-up (T3) we expect a positive outcome in the psychomotor and cognitive development of childrem enrolled in the study. | |
|  | Patient Type | The study sample will consist of infants born preterm that meets the inclusion/ exclusion criteria of the study (see below) and they will be recruited both in Italy and in Denmark.  The recruitment of children in Italy will be carried out mainly by children discharged from the 'Unit of Neonatology of the Azienda Ospedaliera Universitaria Pisana. | |
|  | *Criteri di inclusione* | Inclusion criteria:   - Gestational age ≥ 28 + 0 weeks and 32 + 6 weeks - Corrected age at T0 between 2 and 9 months and reaching the score limit, in relation to corrected age, of the the gross motor skills on the Ages & Stages Questionnaire (ASQ-3); in detail: - score ≥ 25 at ASQ-3, two months (includes those aged between 1 month and 2 months and 30 days) - score ≥ 10 at ASQ-3, 4 months (includes those aged between 3 months and 4 months and 30 days) - score ≥ 5 and 50 to ˂ ASQ-3, 6 months (includes those aged between 5 months and 6 months and 30 days) - score ≥ 10 and 30 to ˂ ASQ-3, 8 months (it includes those aged between 7 months and 8 months and 30 days) | |
|  | *Criteri di esclusione* | Exclusion criteria  - Gestational age <28 weeks or ≥ 33 weeks   - Small for gestational age (SGA) - Presence of brain damage (congenital malformations, hypoxic-ischemic outcomes, intraventricular haemorrhage> 1 degree or all grades of periventricular leukomalacia) - Epilepsy or other seizures - Severe sensory impairment (blindness, deafness) - Other severe non neurological malformations - Enrolment in other experimental studies with rehabilitative purpose | |
|  | *Exit criteria* | - Withdrawal of informed consent  - Onset of epilepsy or photosensitivity  - Clinical worsening | |
|  | *Evaluation of Response and safety of treatment (visits, follow-ups, examinations)* | As previously described the sample will be assessed 4 times T0, T1, T2, T3. These assessments require the use of standardized and specific evaluation tools for the developmental age (see table 1), these tests provide quantitative and qualitative indices of development.  The evaluation of the group of children recruited in Italy will take place at the IRCCS Fondazione Stella Maris, while the children recruited in Denmark will be evaluated at the HEC.  Operators involved in the clinical evaluations at the two clinical centers (HEC and FMS) have carried out trainings and meeting to obtain a 100%agreement in scoring.  **Primary outcome measure:**  In accordance with the main goal of the study, the Primary outcome measure is   - Bayley III; *Cognitive subscale (T0, T1, T2, T3*)*   The BSID -III are standardized scales for the assessment of the functional development of children from 1 to 42 months of age. These scales allow to identify children with developmental delays and are used and also validated for children born preterm.  In particular the Bayley III cognitive ( Cognitive subscale) is able to evaluate the sensory motor development, the exploration and manipulation, the formation of concepts, memory and problem-solving and other aspects of the cognitive process .  **Secondary outcome measures:**  In order to evaluate the effectiveness of CareToy on promoting postural development, the manual function and the visual development (see objectives) were chosen for the following evaluative scales:  Motor Evaluation:   - *Alberta Infant Motor Scale (AIMS)*; *(T0, T1 e T2)*   It is an instrument validated for the assessment of the motor development, in terms of postural-motor skills, for children up to the 18 months of age .  *- Infant Motor Profile (IMP)*; *(T0, T1 e T2)*  The IMP evaluates the spontaneous motor behaviour of the child in an age between 3 and 18 months, or more precisely until the child has acquired a good experience of independent walking. In short the instrument evaluates not only the motor performance in quantitative terms, but also the qualitative aspects such as the variability and the fluidity of movement and the adaptability of motor strategies. It is a validated instrument both in infants born preterm and at term.  - *CareToy C: evaluation of grasping (T0, T1 e T2)*  The evaluation of the grasping through CareToy C provides the use of bio-mechatronic toys equipped with pressure sensors for quantifying the strength of the grasping and the number of grasping actions (see Description) .    Evaluation of visual function:   - *Teller acuity Cards (T0, T1 e T2)*   The test permits the assessment of the visual acuity also in children under the first year of life. To the child it is shown a board (card) that has on one side a visual target that consists of white / black stripes of different width and on the other one with neutral (gray) background. The test assesses the child's ability to direct the eye to the visual target. The estimation of visual acuity is based on the finest width of stripes that the child fixed and then prefers neutral area. The test is therefore based on the principle of "preferential looking", the indicators of response are based on spontaneous behavioural e.g. eye or head toward the stimulus . The test is of high reliability, it is versatile and requires short execution times.  - *CareToy C: visual evaluation (T0, T1 e T2)*  The visual evaluation through CareToy C provides the use of a system of eye tracker with the purpose of measure the displacement of the gaze and the visual attentional response secondary to the presentation of a visual stimulus (see Description) .  Questionnaires filled in by parents :   - *Ages & Stages Questionnaire*; *(T0, T3):*   The questionnaire is able to assess psychomotor development of children from 1 to 66 months of age. For our study , firstly the area of gross motor skills will be used in order to identify, in each child, the most appropriate time for the starting of the experimental phase by achieving a minimum score (see inclusion criteria) . All the items of the questionnaire will be filled in by the parents atT0 and it will also be repurposed at T3 to monitor the long-term development of the child.   - *Bayley III*; *Social-Emotional subscale* *(T0, T1, T2 and T3):*   It is a subscale of the Bayley III that permit to identify any difficulties in emotional processing and to obtain an indicator of the level of social and emotional development of the child. In particular for assessing the mastery that the child's own emotional functionality, the communication needs, the ability to relate to others, to use emotions in interaction with and finalized and the use of emotional signals to solve problems.   - *Parenting Stress Index (PSI) (T0, T1, T2 and T3)*   This instrument explores the emotional and behavioural dimensions of children and it evaluate the risk for the parent to live his role as dysfunctional.  Table 1   \|  \| **T0** \| **T1** \| **T2** \| **T3** \| \| --- \| --- \| --- \| --- \| --- \| \| **Primary outcome measure** \|  \|  \|  \|  \| \| Bayley III Cognitive Subscale \| ✓ \| ✓ \| ✓ \| ✓^*^ \| \| **Secondary outcome measure** \|  \|  \|  \|  \| \| AIMS \| ✓ \| ✓ \| ✓ \|  \| \| IMP \| ✓ \| ✓ \| ✓ \|  \| \| Teller Acuity Cards \| ✓ \| ✓ \| ✓ \|  \| \| CareToy C measures \| ✓ \| ✓ \| ✓ \|  \| \| **Questionnaires** \|  \|  \|  \|  \| \| Parenting Stress Index \| ✓ \| ✓ \| ✓ \| ✓ \| \| Ages & Stages Questionnaire \| ✓ \|  \|  \| ✓ \| \| Bayley III Social-Emotional Subscale \| ✓ \|  \|  \| ✓ \|  Assessment of compliance to the training: During the experimental training, intervention sessions will be weekly and remotely evaluated by clinical staff in order to:  - assess the evolution of the child  - adapt the training program to the needs of child development.  - assess the compliance of the child to the training  In addition, through a short online interview, the parents will be asked to provide daily and weekly information on the participation and compliance of child training. | |
|  | *Statistical analysis (sample size and method)* | Sample size  The sample size was chosen on the basis of expected changes in the primary outcome measure ( Bayley III, Cognitive subscale, see outcome measures) .  In light of the results emerging from the review of literature, in a study of Mazurek Melnyk et al 2001 , in which it was evaluated the effectiveness of the parent-targeted intervention program (COPE) on cognitive development in preterm children, the response to the change in the cognitive subscale BSID III resulted in a very high effect size between 0.60 and 0.72 at 3 and 6 months of corrected age.  Accordingly to a statistical calculation, considering a alpha level of 0.05 with a power of 80% and an effect of at least 0.6, for our study we would need 45 experimental sessions and 45 control sessions . Moreover, it will be necessary to add a 20% of sessions to cover the possibility of drop-outs . In total will then need to do 108 sessions (experimental and control). Foreseeing the cross-over between the experimental and control group, recruited subjects will be assigned, at different times, to both arms of the study (control and experimental), so for our purposes it will be necessary a sample of 54 children.    The sample will be recruited half (27 subjects) in Italy and half (27 subjects) in Denmark.  The twins will be assigned to the same group in the same time in order to facilitate the parents in the management of the Caretoy system for both babies at the same time.    For more details on the clinical protocol see attach H.  For more details around the clinical protocol see Annex H. | |
|  | *Case report form (CRF)* | Annex A e A1 | |
| Letter for the parents | | Annex B | |
| Written consent for the parents | | Annex C | |
| Letter for the paediatrician | | Annex D | |
| Clinical investigator’s brochure (CIB) o technical description of medical device | | Annexes E1, E2 and E3 | |

| Other comments |  |
| --- | --- |
| Declaration | Approved - conditions / recommendations - Deferred - Rejected |
